# Direct Evidence of the Effect of Water Molecules Position in the Spectroscopy, Dynamics, and Lighting Performance of an Eco‐Friendly Mn‐Based Organic–Inorganic Metal Halide Material for High‐Performance LEDs and Solvent Vapor Sensing

**DOI:** 10.1002/advs.202400879

**Published:** 2024-04-24

**Authors:** Mario Gutiérrez, Mario de la Hoz Tomás, Soumyadipta Rakshit, Luis Lezama, Boiko Cohen, Abderrazzak Douhal

**Affiliations:** ^1^ Departamento de Química Física Facultad de Ciencias Ambientales y Bioquímica e INAMOL Campus Tecnológico de Toledo Universidad de Castilla‐La Mancha (UCLM) Avenida Carlos III, S.N. Toledo 45071 Spain; ^2^ Departamento de Química Orgánica e Inorgánica Facultad de Ciencia y Tecnología Universidad del País Vasco UPV/EHU, B° Sarriena s/n Leioa 48940 Spain; ^3^ Present address: Department of Chemistry Motilal Nehru National Institute of Technology Prayagraj Uttar Pradesh 211004 India

**Keywords:** dual emission, dynamics, self‐trapped excitons, soft material, white LED

## Abstract

Luminescent Mn(II)‐based organic–inorganic hybrid halides have drawn attention as potential materials for sensing and photonics applications. Here, the synthesis and characterization of methylammonium (MA) manganese bromide ((MA)_n_Br_x_Mn(H_2_O)_2_, (*n* = 1, 4 and *x* = 3, 6)) with different stoichiometries of the organic cation and inorganic counterpart, are reported. While the Mn^2+^ centers have an octahedral conformation, the two coordinating water molecules are found either in *cis* (**1**) or in *trans* (**2**) positions. The photophysical behavior of **1** reflects the luminescence of Mn^2+^ in an octahedral environment. Although Mn^2+^ in **2** also has octahedral coordination, at room temperature dual emission bands at ≈530 and ≈660 nm are observed, explained in terms of emission from Mn^2+^ in tetragonally compressed octahedra and self‐trapped excitons (STEs), respectively. Above the room temperature, **2** shows quasi‐tetrahedral behavior with intense green emission, while at temperatures below 140 K, another STE band emerges at 570 nm. Time‐resolved experiments (77–360 K) provide a clear picture of the excited dynamics**. 2** shows rising components due to STEs formation equilibrated at room temperature with their precursors. Finally, the potential of these materials for the fabrication of color‐tunable down‐converted light‐emitting diode (LED) and for detecting polar solvent vapors is shown.

## Graphical abstract

Methylammonium manganese bromide hybrid material is synthesized using different ratios of the organic and inorganic counterparts. The material with the lowest organic content has two cis‐coordinated water molecules resulting in octahedral red‐emitting Mn (II). The one with the highest content, the water molecules are in trans‐position, showing quasi‐tetrahedral behavior with dual green and red emission from free and self‐trapped excitons.

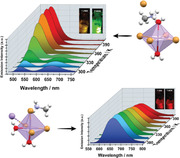

## 1 Introduction

During the last two decades, hybrid organic–inorganic metal halide (OIMH) perovskites have drawn significant attention as potential materials for electronic and optical devices.^[^ ^1^ ^]^ In the early years of their emergence, the research was focused predominantly on the use of lead (Pb)‐based halide perovskites as visible‐light sensitizers in solar cells.^[^ ^2^ ^]^ Over the years, the OIMH perovskites have shown great promise for a wide range of technological applications encompassing photovoltaics (PVs), light‐emitting diodes (LEDs), lasers, transistors, photodetectors, and photocatalysts.^[^ ^1^, ^2^, ^3^ ^]^ Although great progresses have been made in the field of Pb‐halide perovskite‐based photovoltaics, the high toxicity of Pb and the overall low stability of the devices severely hinder their commercial exploitation.^[^ ^4^ ^]^ Therefore, several strategies have been developed to circumvent this issue, such as the partial or complete replacement of Pb with other nontoxic divalent and trivalent cations, e.g., Mn^2+^, Sn^2+^, Cu^2+^, Sb^2+^, Ce^3+^, In^3+^, and Bi^3+^.^[^ ^5^ ^]^

Among these, Mn‐doped and Mn‐based perovskites have emerged as a viable alternative to the Pb‐based ones.^[^ ^5^, ^6^ ^]^ Thus, during the past few years, numerous studies have reported on the synthesis and characterization of these materials. As dopants, the Mn ions can stabilize the perovskite phase and can provide efficient dual‐emission from both the Mn ions (Mn^2+^) and the host material.^[^ ^7^ ^]^ This approach has been used to synthesize Mn‐doped CsPbX_3_ or MAPbX_3_ (MA = CH_3_NH_3_, X = Cl, Br, and I) with superior optoelectronic properties than those of the undoped counterparts.^[^ ^7^, ^8^ ^]^ Interestingly, organic–inorganic hybrid perovskites with only Mn^2+^ ion as the B‐site cation exhibit tunable photophysical properties depending on the coordinating environment of Mn^2+^, since the different crystal field coordination changes the internal structure of the crystal and leads to differences in their optical properties.^[^ ^6b^ ^]^ It is generally accepted that in a hybrid OIMH perovskite structure, tetrahedrally coordinated Mn^2+^ exhibits green emission, whereas in an octahedral coordination, it emits in the red region.^[^ ^6^, ^9^ ^]^ The color of the emission is also related to the Mn–Mn distance between the emitting centers, which is usually shorter (3–5 Å) for the red‐emitting octahedra and significantly longer (6–12 Å) for the green emitting tetrahedra.

So far, the majority of the Mn(II) hybrids present only a single emission band, either green or orange/red, originating from the d−d transition of the Mn(II) units.^[^ ^5^, ^9^, ^10^ ^]^ However, examples of dual‐emissive Mn‐based OIMH perovskites have also been reported.^[^ ^10^, ^11^ ^]^ While detailed studies of these systems have been undertaken, a clear understanding of this extraordinary behavior remains elusive. For one such example, the dual emission was suggested to arise from the coexistence of both tetrahedral and octahedral Mn(II) in a single structure.^[^ ^11a,c^ ^]^ In another example, the dual emission of the 2D hybrids of (C_6_H_5_(CH_2_)_x_NH_3_)_2_MnCl_4_ (x = 1−4) was explained in terms of green emission associated with the organic cations, while the red one was attributed to the ^4^T_1_(G) → ^6^A_1_(S) transition of Mn(II).^[^ ^11b^ ^]^ The C_4_N_2_H_14_MnBr_4_ hybrid with a single Mn site was reported to exhibit a dual emission from green and red bands, corresponding to self‐trapped excitons (STEs) and weak ferromagnetic Mn‐Mn interaction, respectively.^[^ ^11d^ ^]^ [C_9_NH_14_]_2_MnI_4_ and chiral (R‐/S‐Br‐MBA)_3_MnBr_5_ are other examples of the involvement of STEs in the simultaneous red and green emission bands.^[^ ^11g,h^ ^]^ Finally, in a recent work, the dual emission of a new (3‐(dimethylamino)‐propyl)(triphenyl)phosphonium)[MnBr_4_] hybrid was suggested to have the same origin where the green emission is dominant while the red one, considered as a satellite of the former, is caused by lattice distortions and vibrations of the organic linker N‐H group interacting with the bromine atom.^[^ ^11f^ ^]^

The organic ligands participating in and stabilizing the lattice are an important component in tuning and separating the Mn^2+^ coordination units in OIMHs and play a fundamental role in the construction of their electronic band and crystal structures.^[^ ^9^, ^12^ ^]^ Many schemes involving branched organic cations have been developed to control the separation of the Mn(II) emissive units and aim to improve the photophysical properties of the resulting hybrid materials.^[^ ^9^, ^12^, ^13^ ^]^ Surprisingly, while the simplest OIMH perovskite using methyl ammonium (MA) as the organic cation was reported, no photophysical characterization nor possible applications in lighting (LEDs) or photosensing have been performed so far.^[^ ^14^ ^]^ This smaller organic cation allows ease synthesis and may provide a higher flexibility to tune the optoelectronic properties of the resulting OIMH materials by selectively adjusting the ratio of the constituent component in the synthesis. For example, by controlling the amount of the organic cation (pyridine), two different Mn‐based perovskite structures were reported (C_10_H_12_N_2_MnBr_4_ and C_5_H_6_NMnBr_3_ with different spectroscopic and magnetic properties.^[^ ^9a^ ^]^ Thus, using the simplest components to make these materials and their full characterization is of great interest for further development of the Mn‐based OIMH field aiming for a better understanding of their behavior for potential electro‐optics applications.

Herein, we present a systematic synthesis, structural, and detailed spectroscopic study of Mn‐bromide OIMH with MA as the organic cation. We synthesized three samples with different MABr:MnBr_2_ ratios of the two components involved in the synthesis. The obtained crystalline solids show different emission colors depending on the ratio used in the reaction. The OIMH with the lower MA content (**1**, MABr:MnBr_2_ ratio of 1:2) is formed by octahedral Mn(II) centers with 2 water molecules coordinated in *cis* position, and its emission spectrum consists of a single band with the maximum intensity at 650 nm in the whole studied temperature interval between 77 K and 403 K. The time‐resolved experiments on **1** at temperatures above 160 K suggest contributions from two species emitting in the red with decay times of 55 and 150 µs associated with coupled and isolated emitting Mn(II) centers, respectively. On the other hand, the sample with the highest MA content (**2**, MABr:MnBr_2_ ratio of 2:1) presents significantly different behavior. Single crystal X‐ray diffraction (SCXRD) reveals that the Mn^2+^ centers are also in octahedral coordination but the two water molecules are coordinated in *trans* position. This change in the water molecules’ coordination with the Mn ions gives a tetragonally compressed octahedra, which is responsible for the dual‐emissive behavior at room temperature. The resulting red emission in this sample is explained in terms of the formation of STEs. At temperatures below 140 K, a second type of STEs emission is observed with the maximum emission intensity at 570 nm. This STE is associated with additional axial octahedral compression at these temperature ranges as evidenced by the SCXRD data. Interestingly, the time‐resolved emission measurements show that the green and red emission bands at room temperature are connected by a common channel whose dynamics occur in 10–15 µs. At longer times (>100 µs), both excited emitters (free excitons (FE) and STEs) become equilibrated. Furthermore, upon increasing the temperature, **2** only emits bright green light, which we explain in terms of axial Mn─OH_2_ bond elongation to produce a tetragonally elongated octahedral (quasi‐tetrahedral) structure along with quenching of the STE emission. Remarkably, the intermediate sample, **3**, with a MABr:MnBr_2_ ratio of 1:1, exhibits a mixture of the two phases (*cis* and *trans*‐coordinated water) and shows a behavior that combines the photophysical characteristics of both samples **1** and **2**. We further demonstrate that **3**, with its mixed behavior, is an ideal candidate for the fabrication of a down‐converter white light emitting diode (wLED) with a stability of 83% after 9 h of continuous working operation. Finally, as a second proof‐of‐concept, we show that **3** could be used for detecting vapors of a variety of polar and organic solvents. Therefore, the results presented here reflect the potential of using simple combinations of small MABr organic cation and MnBr_2_ inorganic salt to produce on‐demand Mn‐based hybrid compounds of different crystalline structures and photophysical properties for different photonics applications, such as materials to be integrated as robust phosphors for down‐converter LED devices, in which the emission color can be easily tuned with the applied voltage or sensors for changes in the temperature or for detecting volatile polar compounds.

## 2 Results and Discussion

### 2.1 Single Crystal X‐Ray Diffraction

**Figure 1:**
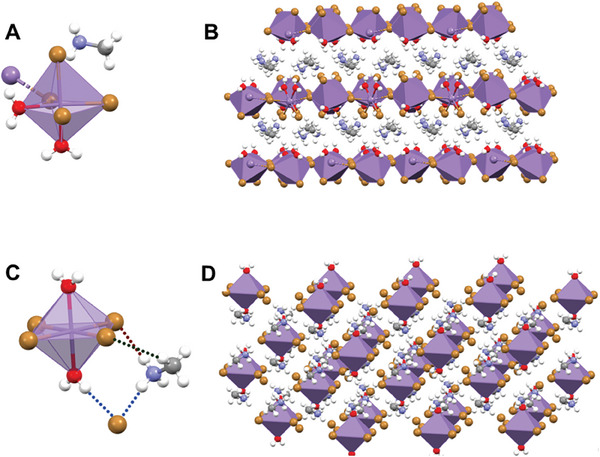
A–D) Illustration of the crystalline structure of the asymmetric part of the unit cell (A) and the packed structure (B) for (MA)Br_3_Mn(H_2_O)_2_ (**1**) and the asymmetric part of the unit cell (C) and the packed structure (D) for (MA)_4_Br_6_Mn(H_2_O)_2_ (**2**). The dotted lines in C indicate the H‐bonding interactions between the methylammonium (MA) and Br (free and coordinated with Mn) and between the H atom of water and the uncoordinated Br. The crystallographic data for MA)_4_Br_6_Mn(H_2_O)_2_ are deposited in the Cambridge Crystallographic Data Centre (CCDC 2323500). The crystallographic data for (MA)Br_3_Mn(H_2_O)_2_ were taken from ref. [14].

To characterize the crystalline structure of the Mn‐based hybrid compounds (Table S1, Supporting Information), we synthesized suitable crystals of **1** and **2** for single‐crystal X‐ray diffraction (SCXRD) analysis. The sample with the smallest molar ratio, **1**, has lattice parameters of a = 7.97 Å, b = 9.59 Å, c = 11.90 Å, α = γ = 90°, β = 91.14°; and crystallizes in the monoclinic phase and P2_1_/c group. This perfectly matches with a reported crystalline structure of molecular formula (MA)Br_3_Mn(H_2_O)_2_.^[^ ^14^ ^]^ This Mn‐based structure is built by 1D chains of [MnBr_2_Br_2_/_2_(H_2_O)_2_]^−^ octahedra (**Figure** **1**A,B), sharing common corners and connected to each other via intermolecular H‐bonds with the MA cations through Br^…^HN, N^…^HO and Br^…^HO bridges. In this structure, the water molecules are coordinated with the Mn center in *cis* position with Mn‐O distances of 2.217(8) Å (O1) and 2.228(8) Å (O2). The distances between the Mn‐Br are not identical and give 2.643(2) and 2.670(2) Å for the terminal Br atoms in *trans* position, and 2.706(2) and 2.726(2) Å for the bridging ones. As a result, the octahedral structure is distorted with X‐Mn‐X angles larger than 90°.^[^ ^14^ ^]^

On the other hand, the compound synthesized with the highest MABr:MnBr_2_ ratio, **2**, shows a crystalline structure not reported hitherto. The SCXRD data were obtained by measuring a crystal of this sample at 80 and 250 K. This Mn‐halide organic–inorganic heteroleptic structure presents a molecular formula of C_4_H_28_Br_6_MnN_4_O_2_ (i.e., (MA)_4_Br_6_Mn(H_2_O)_2_) and crystalizes in the monoclinic phase and C2/m space group. The obtained lattice parameters are: a = 12.0487(4) Å, b = 8.8974(4) Å, c  =  10.2363(4) Å, α = γ = 90°, β = 107.020(4)°, V = 1049.29(7) Å^3^, and Z = 2. Figure 1C illustrates the asymmetric unit of (MA)_4_Br_6_Mn(H_2_O)_2_ along with the packed structure, while the detailed crystallographic parameters are provided in Tables S2–S8 (Supporting Information). In **2**, the Mn^2+^ is hexacoordinated to four Br atoms and two water molecules in an octahedral configuration. The water molecules are coordinated in *trans* position with a Mn−OH_2_ distance of 2.213 (3) Å, while the Mn−Br distances are 2.7078(5) (Br1) and 2.6953(5) (Br2) Å (Table S5, Supporting Information). The Br‐Mn‐Br angles are 180° (Br1−Mn−Br1) and 90.78° (Br1−Mn−Br2) while the O−Mn−Br angles are 90°, meaning an almost ideal octahedra (Table S6, Supporting Information). The Br_4_Mn(H_2_O)_2_ octahedral clusters in **2** are separated from each other by MA linkers, which are intercalated in the structure (Figure 1D). The SCXRD studies of **2** at 80 K (Figure S1 and Tables S9–S16, Supporting Information, CCDC 2339285) demonstrate that the structure remains the same. The data corroborate that the structure resolved at 250 K with a slight decrease in some of the interatomic distances due to the high drop in the temperature. Notably, while the Mn‐Br distances remain unchanged, the Mn−OH_2_ ones are shortened by 0.015 Å from 2.213 Å at 250 K to 2.198 Å at 80 K. Additionally, the shortest Mn‐Mn distance also decreases by 0.057 Å from 7.489 Å at 250 K to 7.432 Å at 80 K. The 3‐D crystalline network of **2** at the two temperatures is stabilized by different intermolecular H‐bonding interactions occurring between the water molecules, MA linker, and Br atoms. It is worth noting, that there are also “uncoordinated” bromine atoms that further stabilize the structure by inducing extra H‐bonding interactions. Particularly, there are four different H‐bonding interactions: 1) O—H^…^Br (H atom from water and uncoordinated Br); 2) N—H^…^Br (H atom from the amine of the MA linker and uncoordinated Br); 3) N—H^…^Br (H atom from the amine of MA linker and Br coordinated with Mn); and 4) C—H^…^Br (H atom from the methyl group of MA linker and Br coordinated with Mn) (Figure 1C).

To date, there are two reported Mn‐based crystalline structures using MA as a spacer.^[^ ^14^ ^]^ The first one also corresponds to a heteroleptic structure where the water molecules are coordinated to Mn in *cis* position (sample **1**), while the second one corresponds to the same sample but with a dehydrated structure where the water molecules are detached from the crystalline Mn‐hybrid material. Both structures can be interconverted by gently heating (formation of dehydrated perovskite) and by exposure to ambient moisture (formation of hydrated sample with water in *cis* position). In these two samples, the Mn centers also present an octahedral configuration, but the Mn‐Mn distances are much shorter (3.333 Å for the dehydrated perovskite, and 4.832 Å for the sample with two water molecules coordinated in *cis* position) than those found in the newly synthesized Mn‐based hybrid material (7.489 Å at 250 K and 7.432 Å at 80 K, sample **2**). Furthermore, while the angles in **2** are almost ideal (Br1‐Mn‐Br1 – 180°, Br1−Mn−Br2 – 90.78°, and O−Mn−Br – 90°), those for **1** deviate from these values to give rise to a distorted octahedral configuration.

Since the Mn‐Mn distance is critical for the emission properties of the OIMH material (longer distances might eliminate the direct spin−spin coupling between Mn ions), we anticipate that the longer Mn‐Mn distances found in **2** will have a direct impact on its emission properties, reflecting the importance of water coordination and the amount of MA spacer to tune the structural and optoelectronic properties of these structures.^[^ ^6b^ ^]^ Furthermore, the two ligands (or spacers) have different field strengths with the Br^−^ being the π‐donating ligand (weak‐field ligand), while the H_2_O one has a more neutral σ‐only nature.^[^ ^15^ ^]^ Therefore, the difference in the ligand field strength coupled with the structural isomerism and the variation in the distance between the Mn centers will govern the spectroscopic and photophysical properties of the resulting OIMH compounds.

### 2.2 Hirshfeld Surface Analysis

**Figure 2:**
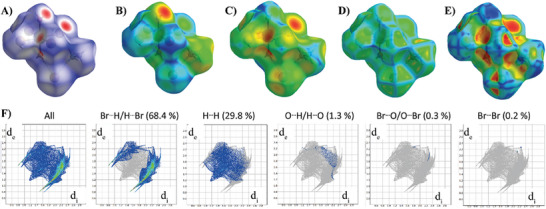
Hirshfeld surface analysis of (MA)_4_Br_6_Mn(H_2_O)_2_ (2) mapped with: A) d_norm_, B) d_i_, C) d_e_, D) curvedness, and E) shape‐index.

To further explore the intermolecular interactions that stabilize the new crystalline structure of **2**, we have generated the Hirshfeld surfaces (HSs) and fingerprint plots (**Figure** **2**A–F). HSs have been mapped with *d*_norm_, *d*_i_, *d*_e_, shape index, and curvedness properties. In this analysis, d_i_ is the distance from the surface to the nearest nucleus included within the surface, while d_e_ is the distance from the surface to the nearest nucleus outside the surface.^[^ ^16^ ^]^ The normalized contact distance (*d*_norm_) is defined by Equation (1):

(1) $$d_{\mathrm{norm}}=\frac{\left(d_{i}-r_{i}^{vdw}\right)}{r_{i}^{vdw}}+\frac{\left(d_{e}-r_{e}^{vdw}\right)}{r_{e}^{vdw}}$$

being *r*_i_^vdw^ and *r*_e_^vdw^ the van der Waals radii of the internal and outer atoms with respect to the surface, respectively.

The blue sides of the *d*_norm_ HS correspond with regions where the probability of finding intermolecular interactions is rather low, whereas the red spots indicate regions with a high probability of intermolecular interactions. These non‐covalent interactions in **2** mainly correspond to intermolecular H‐bonds between Br and H atoms of different molecular moieties. Particularly, those are attributed to: 1) N–H^…^Br interactions between the methyl amine molecule and “free” Br atom; 2) N–H^…^Br interactions between the methyl amine and the Br atom coordinated to the Mn center; and 3) O–H^…^Br interactions between the water molecule coordinated to Mn and the “free” Br atom. The importance of the H^…^Br interactions in this Mn‐based compound is reflected on the fingerprint maps, showing a strong contribution with a total of 68.4% (Figure 2F). It is worth noting that the second highest contribution is found for H^…^H interactions, appearing in the middle of the map. These are caused by the high number of H atoms on its surface. However, they do not confer extra stability to the structure. Additional interactions are found between H^…^O, Br^…^O, and Br^…^Br atoms, though their contribution is minimal. These results demonstrate the importance of the intermolecular H‐bond interactions on the stability of the crystalline structure of (MA)_4_Br_6_Mn(H_2_O)_2_ (2). We will invoke the relevance of these interactions in the photophysical properties of these samples, like those involving STEs.

### 2.3 Powder X‐Ray Diffraction Results

The crystalline structure of the as‐synthesized OIHMs was also confirmed by powder X‐ray diffraction (PXRD) analysis. The PXRD pattern of **1** at room temperature (RT) is consistent with the simulated one obtained from the reported single crystal data, corresponding to the MAMnBr_3_(H_2_O)_2_ in octahedral configuration with the two water molecules coordinated in the *cis* position with respect to each other (Figure S2A, Supporting Information).^[^ ^14^ ^]^ The PXRD diffractogram of **1** changes significantly upon heating to 373 K (100 °C) and the pattern closely resembles the simulated one for the reported single crystal of the dehydrated perovskite (Figure S2B, Supporting Information). On the other hand, the PXRD pattern of the as synthesized **3** is more complex and indicates the presence of at least two different crystalline structures. Although upon the comparison of the pattern of **3** with the simulated one of the *cis*‐hydrated octahedra, the positions of many peaks coincide, we observe a notable number that are not present in the simulated spectrum (Figure S3A, Supporting Information). A similar observation can be made when we compare the diffractogram with the simulated one for the *trans* water‐coordinated octahedra (Figure S3B, Supporting Information). However, if the PXRD signals of the simulated *cis*‐ and *trans*‐hydrated octahedra are summed, the resulting diffractogram is almost an identical replica of the experimental one obtained for **3** (Figure S3C, Supporting Information). This observation suggests that under the synthesis conditions and the selected molar ratio of MnBr_2_:MABr (1:1), this OIHM is present in two phases (two isomer forms) – an octahedra coordinated with two molecules of water in a *cis* orientation with respect to each other and a second one, where the two water molecules are in a *trans* conformation. As the SCXRD studies show, the former forms a 1D chain sharing common corners with Mn – Mn distances of 4.832 Å, while in the latter, the octahedra are isolated from each other (Mn–Mn distance of 7.489 Å).

**Figure 3:**
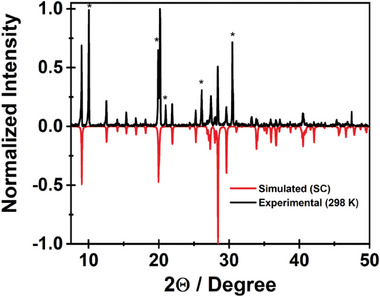
Powder X‐ray diffractograms for **2** at 293 K. The simulated single crystal (SC) diffractogram is shown with a negative sign. The marked peaks (*) correspond to the signal of methylammonium bromide (see Figure S4B, Supporting Information).

Finally, the PXRD pattern of the as synthesized **2** (layered crystals between 50 and 300 µm, Figure S4A, Supporting Information) closely resembles the simulated one from the SCXRD for the *trans*‐coordinated water (**Figure** **3**).

It should be noted that extreme care must be taken during the synthesis of **2** to produce pure material with *trans*‐coordinated water. Minor changes in the ratio of the salts could lead to the presence of traces of **1** producing PXRD diffractogram like the one recorded for **3**. We also recorded the PXRD signal of **2** at 373 K (Figure S4C, Supporting Information). The obtained pattern is significantly different from the one obtained at RT suggesting a change in the crystalline structure of the material. More importantly, it is also very dissimilar to the diffractogram obtained for **1** at 373 K which corresponds to the dehydrated octahedral coordination of Mn(II).^[^ ^14^ ^]^ This discrepancy suggests that at high temperatures, while maintaining its high crystallinity, upon disrupting the interaction of the Mn center with the water molecules, the two samples, **1** and **2**, produce different Mn(II) coordination environments. For **1**, the single crystal data demonstrates that the distance between the Mn ions is reduced (from 4.832 to 3.333 Å) upon increasing the temperature, and the coordination remains octahedral. On the other hand, although no single crystal structure could be obtained for **2** at high temperature, the PXRD diffractogram at 373 K most probably corresponds to Mn(II) in a tetragonally elongated octahedral coordination environment, where the water molecules are further separated (although still present) from the Mn center (stronger quasi‐tetrahedral character).

### 2.4 Electron Paramagnetic Resonance

**Figure 4:**
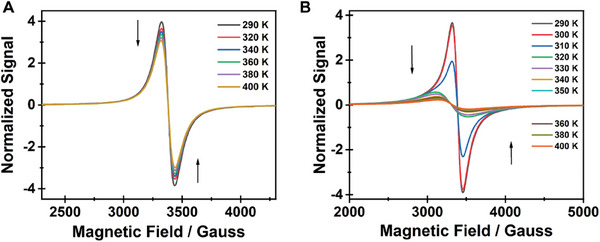
Temperature dependence of the electron‐spin paramagnetic resonance (EPR, X band) spectra of A) **1** and B) **2**.

The EPR spectrum (X band) of **1** at RT shows a single Mn^2+^ signal, characterized by a value of *g* = 2.007 and a “peak‐to‐peak” line width Δ*H*pp = 118 Gauss (**Figure** **4**A). The signal is isotropic and can be fitted well with a Lorentzian‐type line. In similarity with other reports on Mn‐based perovskites, we did not observe the manganese nuclear hyperfine splitting.^[^ ^10a^ ^]^ This behavior is explained in terms of the presence of dipolar interactions in solid samples that lead to a broadening of the lines resulting in a missing hyperfine structure in the spectrum. These types of signals are characteristic of extended systems with significant magnetic interactions between Mn^2+^ ions, these being normally in octahedral coordination and in slightly distorted environments.^[^ ^10^, ^17^ ^]^ Next, when the temperature is increased from RT to 400 K, the intensity of the EPR signal decreases slightly, in accordance with what is expected for a paramagnetic system when the thermal disorder increases, but no change was observed in either the shape or the position of the spectrum. Notably, when the temperature is lowered again to RT, the signal recovers its original intensity.

The EPR spectrum of **2** at RT cannot be fitted considering a single Lorentzian line (Figure 4B). It can be fitted by the sum of two signals – a Lorentzian with values of *g* = 2.054 and ΔHpp = 433 Gauss, which differ significantly from those obtained for **1**, and a narrower signal with values like those obtained for **1** giving *g* = 2.0035 and ΔHpp = 133 Gauss. When the sample is heated to 400 K, this last contribution disappears and does not reappear when the temperature is lowered back to RT, which suggests the presence of a secondary hydrated octahedral phase with water molecules coordinated in *cis* position. The *g* value of the main signal (2.054) is higher than the one usually observed in Mn^2+^ compounds and implies that there is an important orbital contribution to the magnetic moment from excited states.^[^ ^18^ ^]^ This fact along with the large line width suggests a greater distortion of the manganese environment in this compound compared to that of **1**. Finally, preliminary magnetic susceptibility measurements, show that the magnetic interactions in this compound are very weak, which prevents the signal from being narrowed by exchange.

Therefore, while for **1** the EPR spectrum shows a single Mn^2+^ signal corresponding to octahedral environment that does not depend significantly on the temperature, for **2** the spectrum suggests the presence of two phases: one that partially acquires octahedral coordination and a second one consistent with the tetrahedral environment. Upon increasing the temperature to 400 K, the signal shows predominant tetrahedral coordination characteristics, which is consistent with the SCXRD and PXRD data that also indicate quasi‐tetrahedral properties for **2** at higher temperatures.

### 2.5 Thermogravimetric and Differential Scanning Calorimetry

The thermal stability of **1** and **2** was explored through simultaneous thermogravimetric analysis (TGA) and differential thermal analysis (DSC) measurements from RT to 1173 K (900 °C) (Figure S5, Supporting Information). Both curves show that **1** is stable up to 523 K (250 °C) and above this temperature it decomposes in two main stages (Figure S5A, Supporting Information). The TGA curve of **1** shows an initial weight loss of ≈1.2% at temperatures 363–373 K (90–100 °C) which corresponds to the loss of surface‐adsorbed or weakly interacting water. This observation agrees with the reported SCXRD studies that demonstrate loss of water when the sample is gently heated to 100 °C. This loss of water results in the formation of 1D chains with a shortening of the distance between the Mn centers (from 4.832 to 3.333 Å) while maintaining octahedral coordination. The next weight loss, occurring between 493 and 673 K (220 and 400 °C), is attributed to the decomposition of the organic moieties. The observed weight loss in this step is 26.7%, which is comparable to the expected value of 30.4%. This decomposition process is accompanied by a single endothermic peak on the DSC curve (red curve), with the maximum at 636 K (363 °C). The last transformation between 673 and 1053 K (400–780 °C), with a total weight loss of 68.9%, corresponds to the partial decomposition of the inorganic MnBr_2_ unit. Sample **2** is also stable up to 523 K (Figure S5B, Supporting Information). The first weight loss of 60.2%, corresponding to the loss of the organic linkers, is observed in the TGA curve at 523 K and is comparable to the expected one (64.1%). A second weight loss of 37.3%, associated with the inorganic decomposition, appears between 873–1073 K (600 and 800 °C). These two steps are concomitant with two endothermic peaks in the DSC curve. Additionally, another endothermic peak is observed at 426 K (153 °C) which does not correspond to a weight loss change in the TGA curve. The presence of this peak indicates that the sample undergoes a significant phase transition at this temperature. Furthermore, this phase transition is also corroborated by the significant change in the PXRD pattern of **2** at 373 K, which further indicates the soft nature of these Mn‐based materials arising from the coordination of heteroleptic ligands. This conclusion is also supported by the decrease in the interatomic distances in **2** when the temperature is decreased to 80 K as reflected by the SCXRD results. These results agree with those observed for the thermal degradation of similar perovskite compounds.^[^ ^19^ ^]^ More importantly, both samples (**1** and **2**) show high stability with no significant weight loss in the temperature ranging between 298 and 423 K (25 and 150 °C).

### 2.6 Steady–State and Time‐Resolved Properties

#### 2.6.1 Steady–State Absorption and Emission Behavior

**Figure 5:**
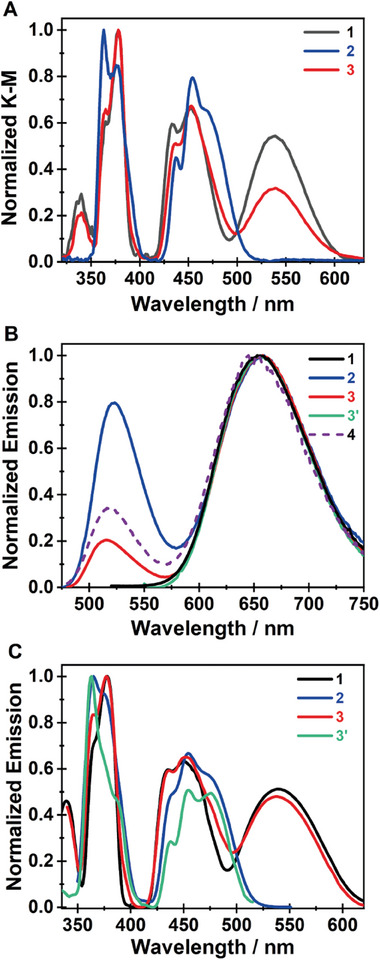
Room temperature and normalized A) diffuse reflectance (converted to K‐M), B) emission, and C) excitation spectra of **1** (black), **2** (blue), **3,** and **3′** (red and green, respectively) samples. The excitation wavelengths were 460 nm for **1**, **2**, **3** (red) and **4** (dashed line), and 530 for **3′** (green). The observation wavelengths were 650 nm for **1** and **3** (red), and 550 nm for **2** and **3′** (green).

To understand the photobehavior of the samples, we recorded UV–vis diffuse reflectance (absorption), emission, and excitation spectra of **1, 2,** and **3** at room temperature (**Figure** **5**). The three samples show strong absorption bands in the UV and 450 nm region for both the reflectance and excitation spectra. For **1**, the first band in the UV region consists of two peaks at 365 and 377 nm and they correspond respectively to the ^6^A_1_ → ^4^E_2_(D) and ^6^A_1_ → ^4^T_2_(D) transitions, whereas the peaks in the visible region at 435, 452, and 467 nm are ascribed to the G‐terms with ^6^A_1_ → ^4^A_1_(G), ^6^A_1_ → ^4^T_2_(G) and ^6^A_1_ → ^4^T_1_(G) transitions (Figure 5A). Additional bands at 337 and 538 nm are observed, which we assign to ^6^A_1_ → ^4^T_1_(P) and ^6^A_1_ → ^4^T_1_(G), respectively. This spectral behavior and specifically the later transitions are characteristics of Mn^2+^ in an octahedral crystal field^[^ ^9^, ^11^ ^]^ and this is in agreement with the reported structure for **1**, formed by 1D chains sharing common corners with Mn – Mn distances of 4.832 Å at room temperature.^[^ ^14^ ^]^

For **2**, the spectrum also exhibits two distinct absorption band groups centered at 375 and 454 nm (Figure 5A). We assign the peaks in the UV group at 363, 376, and 390 nm to the ^6^A_1_ → ^4^T_1_(P), ^6^A_1_ → ^4^E_2_(D) and ^6^A_1_ → ^4^T_2_(D) transitions, respectively. The other three bands in the visible region at 437, 454, and 471 nm related to the G term are ascribed respectively to ^6^A_1_ → [^4^A_1,_^4^E(G)], ^6^A_1_ → ^4^T_2_(G) and ^6^A_1_ → ^4^T_1_(G) transitions. However, **2** compared to **1** does not show any relevant absorption at ≈340 nor at 550 nm, typical of an octahedral configuration of Mn^2+^. Thus, the diffuse reflectance spectrum resembles the one typically associated with the Mn^2+^ in a tetrahedral coordination environment.^[^ ^9^, ^10^, ^19^ ^]^ However, the SCXRD and PXRD data indicate an octahedral environment with four bromine atoms and two water molecules coordinated in *trans* position (Figures 1C and 3). Therefore, although the coordination environment of the Mn^2+^ with *trans*‐coordinated water is octahedral in nature, the long distance between the Mn centers (7.489 Å instead of 4.832 Å in **1**) and the shorter axial Mn‐O bonds with the water molecules (≈2.2 Å) in comparison with the lateral Mn‐Br ones (≈2.7 Å) give rise to a tetragonally compressed octahedral configuration that shows tetrahedral‐like (quasi‐tetrahedral) spectral behavior, contrary to **1**. This quasi‐tetrahedral behavior is further favored by the nature of the ligands, with the two water molecules that occupy the axial positions in **2** being neutral σ‐type (Figure 1C). Notice that for **2**, the SCXRD data indicates the presence of free bromine atoms establishing H‐bond with the coordinated water molecules. These specific interactions, as revealed by the HS analysis and not present in **1**, should induce an electric field around the Mn centers different from the one in **1**, which might provide additional stabilization of the quasi‐tetrahedral behavior of **2**. Furthermore, earlier works on transition metal coordination complexes have shown that it is possible to distinguish between different structural isomers based on their absorption/diffuse reflectance spectra.^[^ ^20^ ^]^ For the general octahedral coordination case, [MA_4_B_2_], where M = transition metal cation, A = ligand 1, and B = ligand 2, it was demonstrated that if A is located to the right of B in the spectrochemical series (stronger field), then for the split band of the *trans*‐[MA_4_B_2_] complex the long wavelength component will be more intense than the short one. On the contrary, if A is placed to the left of B, the shorter wavelength component will be more intense.^[^ ^20^ ^]^ This effect has been documented for CoA_4_B_2_ complexes.^[^ ^20b,c^ ^]^ For the Mn complexes of this type, a similar effect can be expected.  For example, for the *cis*‐ and *trans*‐BrMn(CO)_2_dppm(P(OPh)_3_), where dppm = PhZPCHZPPh_2_, the 530 nm band is present in the *trans*‐isomer and is missing in the one recorded for the *cis*‐isomer.^[^ ^21^ ^]^ For the system under study here, the observed dependence is the opposite, i.e., the *cis*‐isomer is the one showing the 530 nm band. This is in agreement with the general rule since Br^−^ (ligand A in the general structure) is located to the left (weaker field) of H_2_O (ligand B) in the spectrochemical series.^[^ ^21^ ^]^

We also recorded the absorption spectrum of **3** (Figure 5A). This sample was synthesized using a 1:1 stoichiometry of the components, and it is composed of both the green (**2**) and red phases (**1**) (Table S1, Supporting Information). While the diffuse reflectance spectrum of **3** is different from that of **2**, it is resembling that of **1**. The transitions of the three families have their peaks at 340, 364, 378, 436, 453, 468, and 538 nm. They have the same energies of **1** and are characteristics of the octahedral coordination environment of the Mn centers.^[^ ^9^, ^10^ ^]^ Notice that the band intensities at 436 and 538 nm for **3** are almost an average of those of **1** and **2**.

Now, we discuss the steady‐state emission spectra of the three samples. To begin with, **1** shows a red emission, while **2** and **3** give a yellow one (Table S1, Supporting Information). Figure 5B shows that the emission spectrum of **1,** independent of the excitation wavelength, consists of a single band with the maximum emission intensity at 660 nm, while those of **2** and **3** exhibit a dual emission with intensity maxima at 522 and 660 nm. The red emission bands of the three samples are very similar in position and shape. Interestingly, while the dual emission of **2** does not depend on the excitation wavelength, that of **3** does, thus providing the possibility to get both green and red bands or only the red one. Although the excitation of **3** at 460 nm yields the same dual‐band emission spectrum as the one observed for **2** (note that the intensity of the green band is lower for **3**), its excitation at 530 nm produces an emission spectrum like the one obtained for **1**. The green and red bands in the dual emission of **2** (and **3**) have different full‐width at half‐maximum (FWHM) of their intensities: 1700 and 2200 cm^−1^ for the green and red bands, respectively, suggesting larger spectral relaxation of the red emitters or the presence of more than one emitting population. Finally, we recorded the emission spectrum of **4** (excitation at 460 nm), which corresponds to 1:1 ratio (by mass) mechanical mixture of **1** and **2** (Figure 5B). The resulting emission spectrum closely resembles the one recorded for **3** under the same excitation and recording conditions with the notable difference being that for **4,** the intensity of the band at 520 nm is slightly higher than the one for **3**. This difference suggests that the initial ratio of 1:1 of the organic and inorganic salts used for the synthesis of **3** does not necessarily produce 1:1 ratio of *cis* and *trans* isomers.

The excitation spectra of **1** and **2** are comparable to the corresponding reflectance ones and do not depend on the observation wavelength, while those of **3** depend on the gating wavelength and therefore are different from the reflectance spectrum (Figure 5C). The one collected at the red emission maximum (650 nm) closely follows the one corresponding to **1**, while the one at the green band (550 nm) shows the characteristic features of the excitation spectrum of **2**. This observation shows that **3** has two different absorbing phases, that emit in different regions, contrary to **1** and **2** which have a single ground state population, in agreement with the PXRD data. The excitation spectra of **4** (not shown) collected at 520 and 650 nm closely resemble those recorded for **3** under the same experimental conditions.

**Scheme 1:**
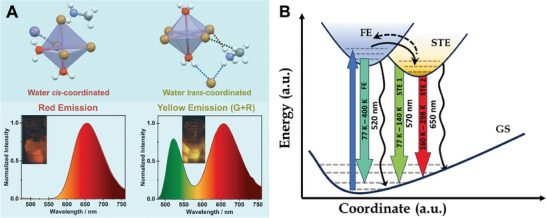
A) Presentation of the octahedral structures of **1** and **2** and their corresponding emission spectra at room temperature. The insets show the emission color of the samples under 365 nm irradiation. B) Schematic presentation of the processes involved in the relaxation dynamics of photoexcited **2** (not to scale). STE: Self‐trapped excitons, FE: free excitons, GS: ground state. The black curved arrows indicate non‐radiative relaxation, while the dashed arrow corresponds to the de‐trapping process.

Now, we assign the origin of the green and red emission in all the samples (**Scheme** **1**). The red emission of **1** is ascribed to the d–d (^4^T_1_ → ^6^A_1_) transition in the octahedral configuration of Mn^2+^emitters coordinated to four bromine atoms and two water molecules in *cis* position. While the green emission band of **2** is characteristic of tetragonally coordinated Mn^2+^ ions, the SCXRD data of **2** shows an octahedral configuration with four bromine atoms and two water molecules coordinated in *trans* position. Therefore, it is likely that for the two structural isomers **1** (*cis*) and **2** (*trans*), the relative positions of the coordinated ligands (Br^−^ and H_2_O) in the spectrochemical series determine their photophysical properties. Similar observations have been reported for other structural isomers of transition‐metal coordination complexes.^[^ ^20^ ^]^ On the other hand, the red emission for **2** suggests that in addition to the free excitons (FE), we also observe the presence of self‐trapped excitons (STEs), as reported for other perovskite materials, including Mn‐based ones, showing dual emission.^[^ ^11^, ^22^ ^]^ STEs can be regarded as excited‐state defects. Upon electronic excitation, and because of the strong coupling between the electrons and phonons, a transient elastic lattice distortion might occur.^[^ ^22^, ^23^ ^]^ Following this photoactivation, the excited electrons are immediately trapped by the deformed lattice releasing energy through recombination and giving rise to a large Stokes‐shift and broadband emission in the reddest spectral region. Notably, the self‐trapping of excitons does not exhibit saturation at high excitation intensities because it is not limited by the concentration of defects.^[^ ^23^, ^24^ ^]^ The mechanism involving STEs emission in **2** is supported by the comparable excitation spectra collected at the maximum emission intensity of both bands along with the lack of the 530 nm absorption band, a typical signature of the octahedrally coordinated Mn^2+^ in these spectra (Figure 5C). From the wavelength values of the maximum emission intensities of the green (522 nm) and red (660 nm), we estimate an energy relaxation of STEs by 4000 cm^−1^ when compared to the Mn^2+^ FE. This value is slightly larger than those (≈3200–3500 cm^−1^) reported for other dual emissive Mn(II) OIHM.^[^ ^11^ ^]^ As the HS analysis shows, several H‐bonding interactions are present in the structure of **2**, which might further affect the efficiency of STE formation. Therefore, while the ligands in **1** and **2** are identical, the different conformations of the coordinating water molecules and the different distances between the Mn^2+^ centers are determining factors for the observed different photophysical behavior. The quasi‐tetrahedral (or hydrated tetrahedral) configuration with two *trans*‐coordinated water molecules has been also observed in the crystal structure of Rb_2_MnBr_4_(H_2_O)_2_ and in C_6_N_2_H_16_MnBr_4_(H_2_O)_2_.^[^ ^25^ ^]^ However, in the former structure, the Mn^2+^ forms differently (from the one reported here) distorted octahedrons that share an edge with [RbH_2_OBr_8_]^7−^, while in the latter, the process of hydration and coordinating the two water molecules in *trans* position is different. The difference in the geometry of the octahedron is sufficient to render different properties to Rb_2_MnBr_4_(H_2_O)_2_, which is not emissive at room temperature, but becomes red emissive at increased temperature and finally emits green light when the water molecules leave the coordination sphere at ≈473 K.^[^ ^25b^ ^]^ Other perovskite systems also have shown a strong relation between the octahedral/tetrahedral distortion and the efficiency of the formation of STEs.^[^ ^10^, ^26^ ^]^ Finally, the photoluminescent quantum yields (PLQYs) of **1**, **2**, and **3** at RT and upon excitation at 450 nm are ≈4%, 6%, and ≈5%, respectively. These low values indicate the presence of additional non‐radiative processes in these soft materials.

#### 2.6.2 Time‐Resolved Emission Decays

**Figure 6:**
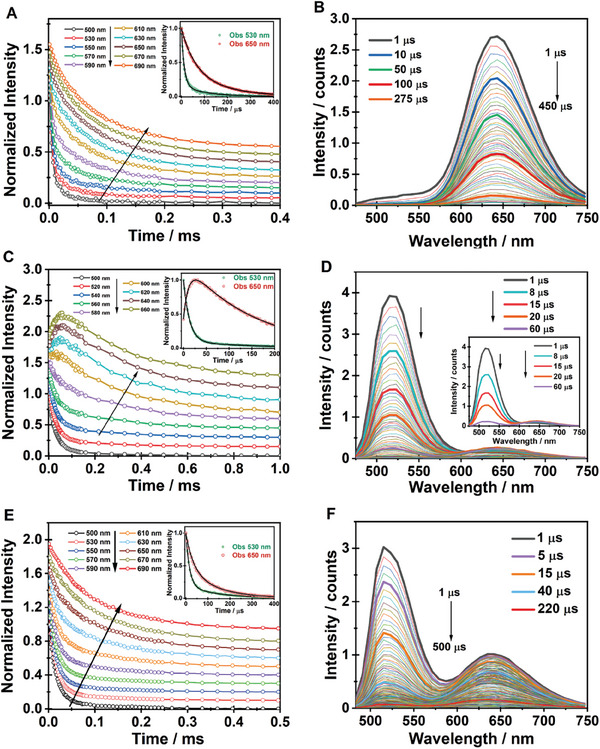
Emission decays collected at selected wavelengths (A, C, and E) and time‐resolved emission spectra (B, D, and F) following excitation at 371 nm for **1**, **2**, and **3**, respectively. The decays are offset (*y*‐axis) for clarity of presentation. The insets in panels A, C, and E show a zoom for the decays collected at 530 and 650 nm. The inset in panel D shows only the highlighted spectra.

To get a deep insight into the photobehavior of these hybrid Mn materials, we recorded emission decays at selected wavelengths and time‐resolved emission spectra (TRES) of the three samples upon excitation at 371 and 433 nm (**Figure** **6**; Figure S6, Supporting Information, respectively). To begin with **1**, independently of the excitation, all the transients at the main red emission band decay biexponentially with time constants of τ_1_ ≈ 55 µs (54%) and τ_2_ ≈ 150 µs (46%) (Figure 6A and **Table** **1**A; Table S17A, Supporting Information). However, when the signal is collected at 500–530 nm (weak emission) the short lifetime is now ≈6–15 µs, notably shorter than the one observed for the rest of the decays. We assign this component to the presence of traces of the *trans* isomer giving rise to the short‐living species in the green emission band. It should be noted that the population of this *trans* isomer is probably very low since its presence is not readily detected in the PXRD pattern of **1**. At the main red emission band, the 50–60 µs component most likely originates from Mn–Mn interacting pairs, while the longer one most probably arises from a population of non‐interacting Mn^2+^ ions. Similar behavior has been observed for the red‐emitting C_5_H_6_NMnCl_3_, where the authors reported a biexponential decay with time constants of 5 and 169 µs.^[^ ^9b^ ^]^

**Table 1:** Values of time constants (τ_i_) and normalized (to 100) pre‐exponential factors (a_i_) obtained from the best fit of the RT emission decays recorded at selected wavelengths upon excitation of **1** (A), **2** (B), and **3** (C) at 371 and 433 nm.

Table 1A	*λ_EX_* = 371 nm				*λ_EX_* = 433 nm			
*λ_obs_/nm*	τ_1_/µs	a_1_/%	τ_2_/µs	a_2_/%	τ_1_/µs	a_1_/%	τ_2_/µs	a_2_/%
500	7	84	62	14	6	43	66	57
530	15	80	113	20	12	43	96	57
650	56	54	148	46	61	57	145	43
725	56	53	148	47	61	58	145	42

Table 1B	*λ_EX_* = 371 nm				*λ_EX_* = 433 nm			
*λ_obs_/nm*	τ_1_/µs	a_1_/%	τ_2_/µs	a_2_/%	τ_1_/µs	a_1_/%	τ_2_/µs	a_2_/%
500	15	84	145	16	13	86	145	14
530	15	84	145	16	13	86	145	14
650	15	−100	148	100	11	−100	147	100
725	15	−100	148	100	11	−100	147	100

Table 1C	*λ_EX_* = 371 nm				*λ_EX_* = 433 nm			
*λ_obs_/nm*	τ_1_/µs	a_1_/%	τ_2_/µs	a_2_/%	τ_1_/µs	a_1_/%	τ_2_/µs	a_2_/%
500	17	78	145	22	16	88	141	12
530	17	78	145	22	16	88	141	12
650	34	54	135	46	55	48	141	52
725	34	54	135	46	55	38	141	62

For **2,** the emission decays and the obtained time constants do not depend on the excitation wavelength, while they strongly depend on the observation one (Figure 6C; Figure S6C, Supporting Information; Table 1B; Table S17B, Supporting Information). At the green band, the signal decays bi‐exponentially with time constants of τ_1_ = 12–15 µs (86%) and τ_2_ = 148 µs (14%). On the other hand, the transients collected at the red band show a single decay component of ≈150 µs along with a rising one of ≈15 µs. The time constant of the latter is comparable to the short decaying one observed at the green emission band, which indicates a common channel between the green and red emitters. In agreement with the discussion in the steady‐state observation for **2**, we assign this component to STEs formation at the excited Mn^2+^, while the long‐lived decay that has its major contribution in the red emission band arises from the relaxation of the equilibrated system. The similar value for the longer decay time at the green and red emission bands further supports the conclusion for an equilibrated STE process with a low energy barrier. While several studies have reported on dual emissive Mn‐based perovskites,^[^ ^10^, ^11^ ^]^ often associated with the presence of two crystalline structures, only a few have demonstrated dual emission behavior associated with the formation of STEs in a single phase.^[^ ^10^, ^11^ ^]^ Here, we also gauged the possibility of an energy transfer from the *trans* isomer phase (green) to remnants of the *cis* one (red), as well as the presence of trap states. However, the steady–state excitation spectra collected at both the green (530 nm) and red (660 nm) emission bands do not show the presence of Mn centers in an octahedral coordination environment where the water molecules are in the *cis* position. We also believe that the red emission does not originate from trap/defect states since the intensity of the emission decays recorded at 650 nm increases linearly with the excitation power (up to 5 mJ), while the associated time constants remain largely unaffected (Figure S6D, Supporting Information). Importantly, the slope has a value of ≈1.1 which is a characteristic value for FE and STE transitions (values between 0.7 and 1.5).^[^ ^27^ ^]^

The time‐resolved behavior of **3** further confirms the mixed nature of this sample (Figure 6E; Figure S6G, Supporting Information). The time constants from the best fit of the decays upon excitation at both wavelengths give values as averages of those observed in **1** and **2,** with the observation that the red‐rising component recorded in **2** is mathematically canceled by the fast red decay of **1** (Table 1C; Table S17C, Supporting Information).

#### 2.6.3 Time‐Resolved Emission Spectra

To further decipher the emission decays, we recorded TRES upon excitation at 371 and 433 nm, and gating in the microsecond regime. The TRES of **1** at both excitation wavelengths consists predominantly of a single band centered at ≈640 nm that decays to almost zero in ≈500 µs (Figure 6B; Figure S6B, Supporting Information). Upon excitation at 371 nm, at early times of observation, we also observed a weak additional band at ≈520 nm. This band decays in the first 10–20 µs, in agreement with the data in Table 1A from the analysis of discrete emission decays in this region, and is most probably associated with the presence of traces of the *trans* isomer giving rise to a change in the crystal field. The TRES of **2** presents more complex behavior that is similar for both excitation wavelengths (Figure 6D; Figure S6E, Supporting Information). The spectra exhibit two bands: one at ≈520 nm that decays in the first 60 µs to a constant signal and persists for longer times (up to 300 µs), and a second one, at ≈660 nm, that forms within the first 60 µs and decays to a constant signal in the same time scale as the one at 520 nm, indicating the conversion of FE (green) to STE (red) and the establishment of an equilibrium between these species at longer timescales. This is also evident by: a) the comparison of the spectra at longer time delays (>100 µs) where both bands retain the same ratio of the emission intensity, and b) the spectrum collected at 160 µs coincides with the steady state one (Figure S6F, Supporting Information). This behavior indicates a reversible process with a low energy barrier between FE and STE. Notice also, the presence of a clear iso‐emissive point at ≈620 nm within the first 60 µs suggesting a coupled and equilibrated process at the excited state (inset Figure 6D). The TRES of **3** recorded upon excitation at 371 or 433 nm presents a dual emission (Figure 6F; Figure S6H, Supporting Information). However, we cannot see any common channel connecting the green and red emitters as we observed in **2**, nor an iso‐emissive point. We explain this discrepancy in terms of mixed antagonist contributions from both **1** and **2**, which masks the process of producing STEs in the green phase of **3**.

### 2.7 Thermochromism

**Figure 7:**
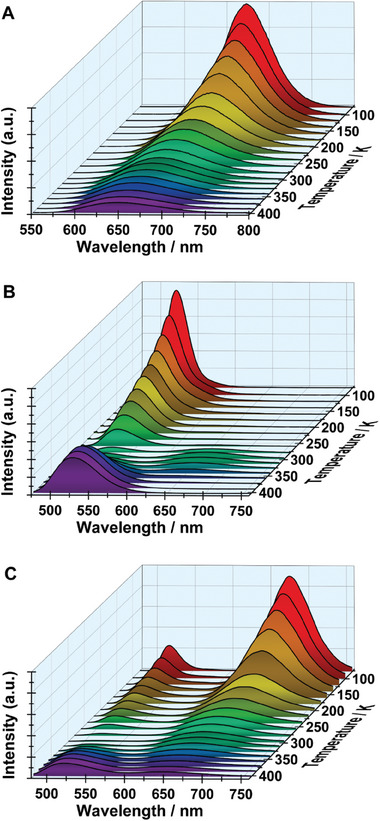
Temperature dependence of the emission spectra of A) **1**, B) **2**, and C) **3**, upon excitation at 450 nm, and collected at the indicated temperatures.

Next, we show and discuss the temperature‐dependent photoluminescence behavior of all the samples in the range between 77 and 403 K. **Figures** **7**A and S7 (Supporting Information) exhibit the temperature‐dependent emission behavior of **1** at different excitation wavelengths. The result is consistent with the reported changes in the single crystal structure with the temperature.^[^ ^14^ ^]^ Upon increasing the temperature, we recorded a decrease in the emission intensity concomitant with a blue shift (30 nm; 680 cm^−1^) of the intensity maximum and an increase in its FWHM from 1343 cm^−1^ at 77 K to 2310 cm^−1^ at 403 K. This behavior is associated with a decrease in the Mn‐Mn distance (from 4.832 Å at RT to 3.333 Å at 373 K for the dehydrated perovskite) due to the loss of the *cis*‐coordinated water as demonstrated by the reported single crystal studies.^[^ ^14^ ^]^ We calculated the activation energy, Δ*E*_a_, associated with this process using Equation (2):

(2) $$I\left(T\right)=I_{0}/\left(1+Ae^{-\Delta E_{a}/k_{B}T}\right)$$

where *I*(*T*) and *I*_0_ correspond to the emission intensity at the experimental and maximum emission intensity temperatures, respectively, *A* is a pre‐exponential factor, and *k*_B_ is the Boltzmann constant. The fit to the temperature dependence of the emission intensity maximum gives a value for Δ*E*_a_ = ≈6.0 kJ mol^−1^, which is comparable to the values reported for other red‐emitting Mn‐based perovskites (Figure S7B, Supporting Information).^[^ ^10a,b^ ^]^ The obtained value is higher than the thermal disturbance energy at RT (2.45 kJ mol^−1^ at 298 K), which reflects the high thermal stability of this type of hybrid material.^[^ ^10b^ ^]^ In the high‐temperature range, upon gradual decrease of the temperature from 403 K back to 303 K, the emission intensity recovers its initial value, which is explained in terms of the re‐adsorption of ambient water molecules to recover the initial Mn coordination state.

The temperature dependence of the emission spectra of **2**, excited both at 360 and 450 nm, follows a more complex behavior (Figure 7B; Figure S8A, Supporting Information). The observed dependence cannot be explained only by the increase in the Mn‐Mn distance, as reported previously for other green‐emissive Mn‐based perovskites.^[^ ^10^, ^19^, ^28^ ^]^ For clarity, we present and discuss the results in three different temperature ranges. The first one comprises the temperature interval between 77 and 140 K (Figure S8A, Supporting Information). To begin with the result at 77 K, the emission spectrum consists of a single and intense band with the maximum emission intensity at 524 nm and a FWHM of ≈1200 cm^−1^. Additionally, it shows a low‐energy tail at ≈570 nm. At this temperature, we estimate (compared areas at 77 K and RT) a PLQY of 36%. Upon increasing the temperature to 140 K, the intensity of the main (green) band decreases steadily concomitant with an increase in the intensity of the 570 nm contribution. We assign the band at 525 nm to the emission of the FE, while that at 570 nm arises from STEs (labeled STE1 for clarity). The thermal quenching of the FE emission to give STEs and possibly non‐radiative states in this temperature range is associated with Δ*E*_a_ = 3.2 kJ mol^−1^. The presence of the low‐energy band and its STE origin agree with previous studies on the low‐temperature photobehavior of Mn‐ and Pb‐based perovskites. For example, low‐energy bands assigned to STEs have been reported for single crystals of MAPbI_3_ in the temperature range between 5 and 298 K.^[^ ^29^ ^]^ Similar behavior was also reported for CsPbX_3_ (X = I, Cl or Br) nanocrystals.^[^ ^30^ ^]^ In these studies, the thermal activation/deactivation transition (between 30 and 100 meV) involving STEs and FE was associated with the deformation of the octahedral axial coordination. Low energy STE exciton bands at temperatures below 100 K have also been demonstrated in C_4_H_14_N_2_MnBr_4_ single crystals and [TMPA]_2_MnI_4_ powder.^[^ ^11d,h^ ^]^ The second temperature range encompasses temperatures between 160 and 298 K (Figure S8B, Supporting Information). Upon increasing the temperature from 160 to 240 K, independently of the excitation wavelength, a new band with a maximum emission intensity at ≈650 nm arises. When the temperature is increased further (Figure S8B, Supporting Information), the intensity of this emission band gradually increases and reaches its maximum at RT. Finally, above RT a decrease in the intensity of this band is observed and then it becomes negligible above 373 K (Figure S8C, Supporting Information). Concurrently, the intensity of the green band (FE) first decreases (160–298 K) and then monotonically increases in the temperature range between RT and 403 K. This also results in a notable upturn in the estimated value of the PLQY that changes by a factor of 5 to reach ≈30%. Based on the temperature dependence of the spectral behavior in the temperature interval between 240 and 403 K, we assign the band at 650 nm to a new type of STEs (denominated STE2 for clarity) with a larger Stokes shift in comparison to the one found at lower temperatures (STE1). For the formation of STE2, we found a sharp change in the activation energy of FE to give STE2 and other non‐radiative states, Δ*E*_a_ = 10 kJ mol^−1^ (Figure S8D, Supporting Information). This clearly indicates that the related process is associated with larger activation energy (3 times) in comparison to the one found for the formation of STE1 (Δ*E*_a_ = 3.2 kJ mol^−1^). When the sample was allowed to slowly cool down to RT, the intensity of the green emission band decreased, while that of the red one recovered its initial value. The recovery of the red emission band (Δ*E*_a_ = 10 kJ mol^−1^) at 650 nm upon cooling down from 400 K is indicative that at RT the related STE process once again is populated and hence the 530 nm emission band corresponding to the FE loses its intensity (Δ*E*_a_ = 32 kJ mol^−1^, Figure S8E, Supporting Information) to give rise once again to the STE2 emission at 650 nm.

The observed temperature dependence of the emission spectrum of **2** along with the lack of the 530 nm absorption band, associated with the absorption Mn^2+^ centers in an octahedral configuration, in the excitation spectra collected at the green and red emission bands further support the presence of STEs both at RT (STE2) and 77 K (STE1). The two STE states observed in the emission spectra of **2** at the low and intermediate temperature intervals show that the electronic band structure is affected by the lattice distortion. Scheme 1B illustrates a generalized picture of the possible processes in **2** involving FEs, STEs, and non‐radiative relaxation at the studied temperatures (77–403 K). Following the optical excitation, a FE is formed and after its thermalization, it becomes trapped in a long‐lived STE state. This trapping is then followed by a Stokes‐shifted broadband emission. A thermally assisted de‐trapping pathway, followed by a non‐radiative relaxation can also be present and plays a key role in the temperature dependence of the PL characteristics. During de‐trapping, the distorted lattice around an STE can revert to its original state through exciton–phonon coupling which depends on the temperature.^[^ ^31^ ^]^ Thus, relatively high temperatures can facilitate de‐trapping and might assist relaxation via a fast non‐radiative channel. At temperatures well above the RT, the self‐trapping process is significantly less efficient since the STEs gain enough energy to efficiently de‐trap and the relaxation of the optically excited system comes mostly through FE emission and non‐radiative decay channels. Two main factors have been invoked to be the origin of the lattice deformation that governs the formation and stability of the STE states.^[^ ^22^, ^23^, ^29^, ^30^, ^32^ ^]^ On one hand, the deformation might arise from distortion of the octahedral configuration of the transition metal center.^[^ ^30a,c^ ^]^ On the other, several studies have demonstrated that the organic cations rigidity affects the lattice deformability and as a result the strength of the exciton‐phonon coupling.^[^ ^31^, ^33^ ^]^ Therefore, for **2**, in the low temperature range, where the lattice is more rigid, a stronger electron–phonon coupling is expected and the observed STE1 should arise from distortions of the Mn^2+^ *trans*‐octahedra, as evidenced by the SCXRD data that show reduced Mn‐O axial distance at 80 K. When the temperature increases, the lattice becomes more locally deformable as the organic cations regain the ability to rotate/vibrate, lowering the exciton‐phonon coupling strength. This process is associated with less efficient STE2 formation, but it also increases the probability of opening new non‐radiative relaxation pathways as evidenced by the sharp decrease in the PLQY of **2** measured at RT. Finally, at temperatures above RT, the excitons gain enough energy to escape (de‐trap) the STE states to give intense FE emission and PLQY of ≈30%.

The temperature dependence of the emission spectrum of **3** follows the mixed nature of the sample observed in the other photophysical studies of this sample (Figure 7C; Figure S9, Supporting Information). When excited at 360 nm (Figure S9A–C, Supporting Information) or at 450 nm (Figure 7C), the trend resembles the one of **2**. However, it should be noted that while the emission spectra of **2** at temperatures above 343 K are only composed of the green band, those for **3** show significant contributions from the red band as well. Similar observation can be made for the low‐temperature range (77–298 K), where the collected spectra maintain the characteristics of both **1** and **2**. The red band at temperatures above 343 K and below 180 K arises from the red phase composed predominantly of the *cis‐*octahedral Mn (Sample **1**). This is further confirmed when we excite **3** at 530 nm with the resulting spectra showing similar behavior to the one observed for **1** (Figure S9D, Supporting Information). The mixed behavior is also reflected in the value of the activation energy (Figure S9E,F, Supporting Information). When it was calculated using the data in the 298–403 K interval, following excitation of **3** at 360 or 450 nm, Δ*E*_a_ = 30 kJ mol^−1^, which is comparable to the one obtained for **2** under the same conditions (≈32 kJ mol^−1^). On the other hand, the temperature dependence of the emission spectrum of **3** following excitation at 530 nm gives Δ*E*_a_ = 6.5 kJ mol^−1^, a value similar to the one obtained for **1** (≈4 kJ mol^−1^).

**Figure 8:**
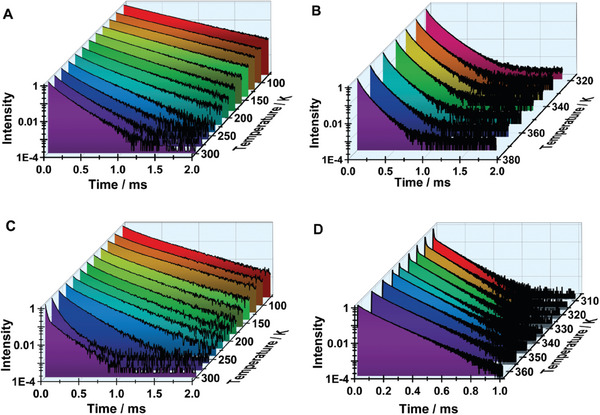
Temperature dependence of the emission decays of A) **1** between 77 and 298 K; **B**) **1** between 313 and 363 K; C) **2** between 77 and 298 K and D) **2** between 313 and 363 K, following excitation at 371 nm.

To further explore the observed emission behavior of **1** and **2** as well as, to support our previous assignment that the red band in the spectra of **2** arises from STE, we studied the temperature dependence of their emission decays at 530 nm in the range between 77 and 363 K (**Figure** **8**; Tables S18–S21, Supporting Information). The emission decays of **1** between 77 and 160 K are mono‐exponential with a time constant of ≈450 µs at 77 K that decreases upon increasing the temperature (Figure 8A; Table S18, Supporting Information). If we assume that the value of the obtained time constant is inversely proportional to the rate constant of the non‐radiative transition (*k*_nr_) and apply the Arrhenius equation, we find that the related process is almost barrierless with *E*_a_ < 1 kJ mol^−1^ in this temperature range. From 180 K, the decays become bi‐exponential and the related time constants (τ_1_ = 126 µs and τ_2_ = 324 µs) keep the trend of gradually decreasing when the temperature increases and reach values of τ_1_ = 43 µs and τ_2_ = 111 µs at 363 K (Figure 8B; Table S19, Supporting Information). This second temperature range (180–363 K) is characterized by a sharp change in the *E*_a_ with a value of ≈4.0 kJ mol^−1^. The overall temperature dependence of the emission decay of **1** can be explained in terms of a more rigid lattice environment at low temperatures. Upon increasing the temperature, the soft material becomes more flexible, which would allow a better coupling between the Mn centers, and as a result, the decay becomes bi‐exponential. Finally, at temperatures above RT, the gradual loss of *cis*‐coordinated water shortens the distance between the Mn centers and allows for the formation of 1D chains that lead to the observed decrease in the two time constants.

In similarity with the steady‐state observations, the temperature dependence of the emission decays of **2** is more complex (Figure 8C,D; Tables S20 and S21, Supporting Information). Here, we present and discuss the results considering three temperature intervals. In the first one, at temperatures below 140 K, the emission transients at 520 nm decay bi‐exponentially, with time‐constants of τ_1_ = 140 µs and τ_2_ = 360 µs. When the emission signal is collected at 570 nm, we observed a rising component (τ_1_ = 180 µs) with a value comparable to the first decay component at 520 nm, which suggests the presence of a coupled process. This process is followed by a decay component of τ_2_ = 490 µs. The presence of a coupled process is further supported by the steady‐state experiments in this temperature range (vide supra), where a second emission band at 570 nm was also observed.

To support this assignment, we also recorded the TRES at 77 K (Figure S10, Supporting Information). The time evolution of the spectra clearly shows the formation of the band at 570 nm at longer gating times. The formation of this band is coupled with the decrease in the intensity of the main band at 520 nm. Therefore, and in similarity with the steady‐state emission spectrum, we assign the rising component of 180 µs to the process of self‐trapping of FE and correspondingly, the band at 570 nm and the component of 490 µs to the emission of STE1. Next, we studied the photodynamic behavior of **2** in the temperature interval between 140 and 298 K. The emission transients collected at the FE band (500 – 550 nm) decay bi‐exponentially with time constants that steadily decrease in value (from τ_1_ = 80 µs and τ_2_ = 293 µs at 140 K to τ_1_ = 15 µs and τ_2_ = 134 µs at 298 K). The relative contribution of the short‐time component increases with the temperature, while that of the longer one decreases. At these temperatures, the decay at 570 nm does not show the long‐rising component observed at lower temperatures. Instead, they follow the same behavior as those at 500 and 520 nm. On the other hand, the decays collected at 675 nm now present a rising component with a value that varies with the temperature and is comparable to one of the short decay components in the FE band (Table S20, Supporting Information). We assign this rising component to the formation of STE2 in agreement with the steady‐state observations. In this temperature range (140–298 K) the Arrhenius plots for the decay and rising time components show a sharp change in the slope that corresponds to *E*_a_ of = 27 kJ mol^−1^. Upon further increase in the temperature, the emission transients collected at 530 nm change their behavior and gradually become almost mono‐exponential with an average lifetime of ≈130 µs at 363 K. In this temperature range, the decays exhibit two trends: 1) the amplitude of the fast component of 15 µs at 298 K, which we associate with the formation of STE2, decreases steadily until it becomes negligible above 345 K (Figure 8D; Table S21, Supporting Information); and 2) the value of the longer decay time 130 µs) is almost temperature‐independent and the transient decays are parallel at longer observation times on logarithmic scale (Figure 8D). This behavior further demonstrates the low energy barrier for the reversible STEs event in **2** and that above ≈345 K the trapped excitons get sufficient energy to escape from the self‐trap states, which results in the observed more efficient green emission concurrent with the complete quenching of the red one. Previous studies on the dual emissive hybrid materials have reported similar temperature dependence of the emission spectra associated with the presence of STEs. For example, a new hybrid compound [TMPA]_2_MnI_4_ was reported to show dual emission at room temperature but presented only intense green emission upon increasing or decreasing the temperature. This behavior was explained in terms of STE emission at RT and quenching of its emission at higher temperatures,^[^ ^11h^ ^]^ Similar behavior has also been reported for the pressure‐induced emission of cesium lead halide perovskite nanocrystals where at higher pressures, more efficient STE formation was observed.^[^ ^34^ ^]^ Therefore, we suggest that due to the lattice deformation of **2** at RT, the excited‐state structural reorganization required to trap a photoexcited excitons is smaller than that for the tetragonally elongated octahedral (quasi‐tetrahedral) high‐temperature phase. Because the STEs formation is mediated by the interaction between the exciton and the lattice distortion, the stronger electron‐phonon coupling in **2** at RT (in comparison to the temperatures above RT) may more effectively bind the photoexcited carriers and make the STEs states (STE2) more optically active giving rise to the red emission band at 650 nm.^[^ ^26^ ^]^ We propose that while at RT the *trans*‐coordinated heteroleptic system is in a tetragonally compressed octahedral configuration (the equatorial Mn‐Br bonds are longer (≈2.7 Å) than the axial Mn‐O ones (2.2 Å)) as evidenced by the SCXRD data, at higher temperatures the structure becomes elongated to further enhance the quasi‐tetrahedral behavior with the axial Mn‐O bonds of the *trans*‐coordinated water molecules becoming longer than the equatorial Mn‐Br ones. Pseudo‐ or quasi‐tetrahedrally elongated configurations of the Mn centers and other transition metal octahedral complexes have been recently reported. For example, two differently Jahn‐Teller distorted octahedral [MnF_6_]^3−^ anions in pseudo‐rhombic and pseudo‐tetragonally elongated configurations have been reported for the K_3_[MnF_6_] compound.^[^ ^35^ ^]^ The properties and stability of such complexes were shown to strongly depend on variations in temperature and pressure. The temperature and pressure effects are manifested in orbital reordering mostly driven by tilts and shifts in the framework, and/or a reconfiguration of A‐site cation ordering.^[^ ^35^, ^36^ ^]^ The proposed mechanism that in **2** the water molecules remain in the Mn coordination sphere is further supported by both the TGA and the DSC curves (Figure S5B, Supporting Information) where we did not observe any indication for a weight loss or phase change in the studied temperature region.

### 2.8 Down‐Converter LEDs

Based on the observed photophysical characteristics, we have leveraged the luminescent properties of the synthesized Mn‐based organic–inorganic hybrid materials to fabricate multicolor down‐converter LEDs. To this end, we have coated a blue (465 nm) LED chip (3.5 × 2.8 mm^2^) with 15 mg of compounds **2** and **3**. The emission properties of these LEDs have been characterized at different applied voltages and forward driving currents.

**Figure 9:**
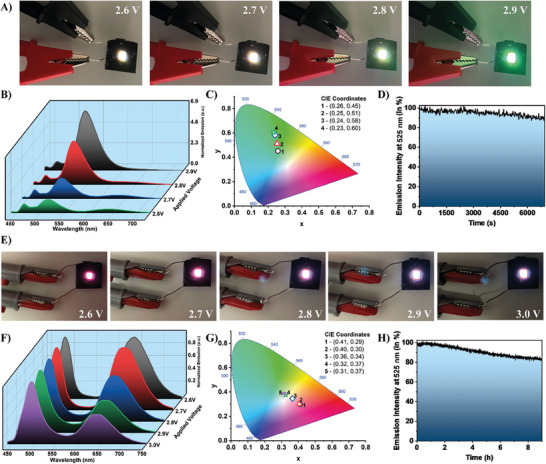
Real photos of the down‐converter LEDs fabricated by depositing 15 mg of **2 (A)** and **3 (E)** onto a blue 465 nm‐LED chip at different applied voltages as indicated in the figures, and corresponding to a forward driving current of 2.6 V → 5 mA, 2.7 V → 20 mA, 2.8 V → 45 mA, 2.9 V → 90 mA, and 3.0 V → 150 mA. Emission spectra of the LEDs fabricated with 15 mg of **2 (B)** and **3 (F)** under different applied voltages. CIE chromaticity coordinates of the LEDs fabricated with 15 mg of **2 (C)** and **3 (G)** under different applied voltages: 1 → 2.6 V; 2 → 2.7 V; 3 → 2.8 V; 4 → 2.9 V; and 5 → 3.0 V. The values of the CIE coordinates for the LEDs with **2** and **3** are respectively: 1: (0.26, 0.45); 2: (0.25, 0.51); 3: (0.24, 0.58); 4: (0.23, 0.60) and 1: (0.41, 0.29); 2: (0.40, 0.30); 3: (0.36, 0.34); 4: (0.32, 0.37); 5 (0.31, 0.37). Representation of the emission intensity collected at 525 nm of the LED prepared with 15 mg of **2 (D)** and **3 (H)** under working operation conditions at an applied voltage of 2.7 V.

The down‐converter LED assembled with 15 mg of **2** emits light from white‐green to deep green upon applying different voltages (**Figure** **9**A). The observed shift in the emission color of the LED is also a consequence of the increase in the emission intensity of the band with a maximum at 525 nm with the applied voltage (increase of the LED temperature, Figure 9B). However, since the emission spectrum of **2** is dominated by the green band, the emission color of the down‐converter LED is primarily green. This is clearly visible when the calculated CIE chromaticity coordinates change from (0.26, 0.45) to (0.23, 0.60) upon increasing the applied voltage (Figure 9C). Finally, the stability of this device was also tested, observing a decrease of just ≈10% of the initial emission intensity after 2 h of continuous working operation (applied voltage of 2.7 V) of the down‐converter LED (Figure 9D).

On the other hand, the emission color of the LED fabricated with **3** shifts from red to white light emission upon increasing the applied voltage (Figure 9E). The observed shift is caused by the decrease in the emission intensity of the red band along with a concomitant increase of the green one (Figure 9F). This change in the emission properties is explained by considering the increment in the temperature of the commercial LED when increasing the applied voltage. For instance, the temperature of the LED device increases with the voltage as: 2.6 V → 304 K; 2.7 V → 310 K; 2.8 V → 319 K; 2.9 V → 336 K; and 3.0 V → 353 K. Hence, these results agree with those explained in the thermochromism section (vide supra). The observed emission colors of the LED devices at different applied voltages closely match with the CIE chromaticity coordinates, where the increase in the voltage shifts the emission from red (0.41, 0.29) to almost cool white light (0.32, 0.37) (Figure 9G). Moreover, we tested the stability of this LED at an applied voltage of 2.7 V (forward driving current of 20 mA). As shown in (Figure 9H), the emission of **3** decreases only by 17% from its initial emission intensity after 9 h of continuous working operation of the commercial LED. Note that the down‐converter LED is not properly sealed (i.e., the Mn‐based compound is exposed to oxygen, moisture, etc.), and therefore, we consider that this loss in the emission intensity makes **3** a promising candidate for developing multicolor emitting LEDs.

### 2.9 Vapochromism

Recent works have demonstrated that by heating or exposing the Mn‐based OIHM to solvent vapors, the photoluminescence can be partially or almost totally recovered. These characteristics have been exploited for the fabrication of rewritable photoluminescent papers and in the sensing of different solvents.^[^ ^10^, ^25^, ^26^, ^37^ ^]^ Therefore, we also tested the sensitivity of **2** to the presence of different solvent vapors. Scheme S1 (Supporting Information) provides a step‐by‐step overview of the vapor sensing process while **Table** **2** gives a summary of the observed behavior. It should be noted that due to the high sensitivity of the prepared paper stripes to the ambient humidity, we could not obtain a reliable emission spectrum of the studied samples. **2** shows increased sensitivity to most of the used polar solvents, such as linear alcohols, tetrahydrofuran, and acetone. Following activation at 55 °C (328 K, intense green emission), the paper stripe emits bright yellow light at room temperature under UV light (365 nm) in agreement with the observed thermochromic properties. Upon exposure to polar solvent vapors for 30 min, the emission is quenched, and the stripe loses its initial coloring. This process is reversible, as upon additional activation at 55 °C (328 K), the paper stripe recovers its initial yellow emission color under UV light (365 nm). A notable case is the interaction of the paper stripe with EtOH, where prior to the complete luminescence quenching, the emission color first changes from yellow to green. We explain the observed sensitivity toward the presence of polar solvent vapors with the disruption/change of the H‐bonded network of the Mn‐based OIHM, which could affect the ligand field, as has been suggested for other vapochromic Mn‐based compounds.^[^ ^10^, ^37^ ^]^ This is further confirmed by the lack of sensitivity toward the vapors of weakly polar solvents such as dichloromethane (DCM), ethyl acetate, and 1,4‐dioxane.

**Table 2:** Behavior of paper stripe impregnated with **2** in the presence of different solvent vapors and under 365 nm‐lamp excitation. The step‐by‐step procedure is given in Scheme S1 (Supporting Information).

	Before activation [Ambient, 25 °C]^a)^	Activation [55 °C]	Before exposure [25 °C, Dry]	After 30 min exposure [25 °C]^a)^	After 50 min exposure [25 °C]^a)^	Recovery [55 °C]	After Recovery [25 °C, Dry]
MeOH, Acetone, THF, 1‐Propanol	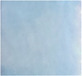	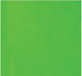	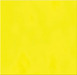	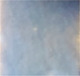	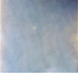	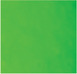	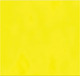
EtOH	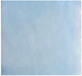	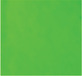	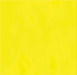	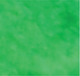	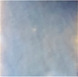	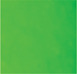	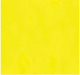
DCM, Ethyl Acetate, 1,4 ‐Dioxane	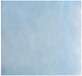	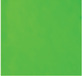	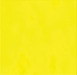	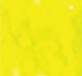	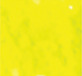	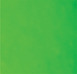	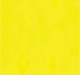

^a)^ The slight blue color comes from the interaction of the paper with the UV light (365 nm).

## 3 Conclusion

In summary, we have successfully synthesized and characterized methylammonium (MA) manganese bromide ((MA)_n_BrxMn(H_2_O)_2_, (n = 1, 4 and x = 3, 6)) using different stoichiometries of the constituent organic and inorganic salts. The SCXRD experiments reveal that in samples with the lowest ((MA)Br_3_Mn(H_2_O)_2_, **1**) and the highest ((MA)_4_Br_6_Mn(H_2_O)_2_, **2**) content of MA, the Mn centers, in an octahedral environment, are coordinated with four bromine atoms and two water molecules, being these later in *cis* and trans position in **1** and **2**, respectively. Sample **3**, with equal stoichiometries of both salt components, is a mixture of **1** and **2**. The different *cis* and *trans* positions of the coordinating water molecules give rise to remarkably different photophysical behaviors. While **1** shows a red luminescence ascribed to an octahedral configuration of the emitting Mn^2+^ centers, **2** exhibits a dual emission spectrum with two bands having the intensity maximum at ≈530 and ≈660 nm, assigned, respectively to Mn^2+^ in a tetraheral‐like configuration and STEs in an octahedral one. The intermediate sample, **3**, gives a mixed behavior of **1** and **2**. The emission decays of **1**, independent of the excitation wavelengths, show two components having time constants of ≈55 and ≈150 µs, assigned to Mn–Mn interacting pairs and non‐interacting Mn^2+^ ions, respectively. A shorter component (7–12 µs) is observed when gating the weak emission, ascribed to a small population of *trans* isomers. For **2**, the emission decays strongly depend on the gated green or red emission. At the former, the signal exhibits two time constants of ≈12 and ≈140 µs, white for the latter we recorded a single decaying component of ≈140 µs along with a rising one of ≈15 µs. The TRES indicates that a population of the green emitters is converted to a red one in ≈15 µs, and after 60 µs, both emitters are equilibrated at the excited state. The short component is assigned to the event of STEs formation at the excited Mn^2+^, while the long‐lived decay that has its major contribution in the red emission band (STEs) arises from the relaxation of the equilibrated system. The RT time‐resolved emission result of **3** is a combination of those of **1** and **2**. We also studied the temperature effect on the steady–state and time‐resolved emission of the 3 samples. While **1** shows a typical temperature dependence of its single emission band, **2** exhibits an interesting temperature effect on its dual emission. Upon varying the temperature from 77 to 403 K, **2** first shows emission from FE at 520 nm along with red‐shifted STE emission at 570 nm (STE1) that shifts monotonically to 650 nm at RT (STE2) due to stronger exciton‐phonon coupling and disappears completely above 343 K. This behavior is concomitant with a strong increase (by a factor of 5) in the intensity of the FE emission. The temperature dependence of the emission of **3** follows that of the mixed **1** and **2** samples and depends on the excitation wavelength. By fine‐tuning the conditions of the synthesis, one can selectively generate materials on demand for a desired photobehavior toward a targeted photonics application. Thus, we show that **2** (and **3**) due to their tuneable dual emission properties can be used as an active layer in potential down‐converter LEDs with a good stability, and selective sensor to the vapor of polar solvents. Therefore, the results presented here should contribute to the advancement of novel low‐cost and eco‐friendly soft organic–inorganic manganese‐based materials with photophysical properties that can be tuned on demand for optoelectronic devices.

## 4 Experimental Section

The Experimental section is described in detail in Supporting Information. It includes the synthesis procedure of methylammonium bromide salt (MABr), and the corresponding Mn‐based OIMH hybrid materials, both as single crystals and crystalline powders. It also describes the techniques and experimental conditions used for the characterization of the studied materials.

CCDC 2323500 and CCDC 2339285 contain the supplementary crystallographic data for this paper. These data can be obtained free of charge from The Cambridge Crystallographic Data Centre via www.ccdc.cam.ac.uk/data_request/cif.]

## Conflict of Interest

The authors declare no conflict of interest.

## Supporting information

Supporting Information

## Data Availability

The data that support the findings of this study are available from the corresponding author upon reasonable request.
