# Supplementary material for: Direct Evidence of the Effect of Water Molecules Position in the Spectroscopy, Dynamics, and Lighting Performance of an Eco‐Friendly Mn‐Based Organic–Inorganic Metal Halide Material for High‐Performance LEDs and Solvent Vapor Sensing
Source: Adv Sci (Weinh). 2024 Apr 24;11(26):2400879. doi: 10.1002/advs.202400879 (PMC11234429; doi:10.1002/advs.202400879)
Supplement: Supplementary file 1 — Supporting Information [file ADVS-11-2400879-s001.pdf]

ADVANCED SCIENCE
Open Access

Supporting Information

for *Adv. Sci.*, DOI 10.1002/advs.202400879

Direct Evidence of the Effect of Water Molecules Position in the Spectroscopy, Dynamics, and Lighting Performance of an Eco-Friendly Mn-Based Organic–Inorganic Metal Halide Material for High-Performance LEDs and Solvent Vapor Sensing

Mario Gutiérrez, Mario de la Hoz Tomás, Soumyadipta Rakshit, Luis Lezama, Boiko Cohen and Abderrazzak Douhal**

Supporting Information

Direct Evidence of the Effect of Water Molecules Position in the Spectroscopy, Dynamics, and Lighting Performance of an Eco-friendly Mn-based Organic-inorganic Metal Halide Material for High-performance LEDs and Solvent Vapor Sensing

Mario Gutiérrez,[1] Mario de la Hoz Tomás,[1] Soumyadipta Rakshit,[1,†] Luis Lezama,[2] Boiko Cohen,[1,] and Abderrazzak Douhal[1,*]*

[1]Departamento de Química Física, Facultad de Ciencias Ambientales y Bioquímica, e INAMOL, Campus Tecnológico de Toledo, Universidad de Castilla-La Mancha (UCLM), Avenida Carlos III, S.N., 45071 Toledo, Spain.

[2]Departamento de Química Orgánica e Inorgánica, Facultad de Ciencia y Tecnología, Universidad del País Vasco, UPV/EHU, Bº Sarriena s/n, 48940 Leioa, Spain.

[†]Present Address: Department of Chemistry, Motilal Nehru National Institute of Technology, Prayagraj, Uttar Pradesh 211004, India

*Correspondence: boiko.cohen@uclm.es; abderrazzak.douhal@uclm.es

Content

Topic	Pages
**Materials and Methods…………………………………………………...**	**S3-S6**
Materials………………………………………………………………	S3
Synthesis of MABr………………………………………………………	S3
Synthesis of Mn-based OIHM……………………………………………	S3-S4
Vapochromism Experiment………………………………………………...	S4
Characterization and Measurements………………………………………..	S4-S6
**Crystallographic data…………………………………………………..**	**S7-S9**
**Time-Resolved Data……………………………………………………**	**S10-S11**
**PXRD figures…………………………………………………………...**	**S12-S13**
**TGA figures……………………………………………………………..**	**S13**
**Time-Resolved Figures…………………………………………………**	**S14**
**Thermochromism Figures……………………………………………..**	**S15-S16**
**Acknowledgments……………………………………………………….**	**S17**
**References……………………………………………………………..**	**S17**

Materials and Methods

Materials:

Manganese (II) bromide tetrahydrate ($MnBr•4H_2O$, 98%) was obtained from Acros Organics (Spain). Ethyl Acetate, Hydrobromic acid (HBr, 48% w/w aq. soln.) and anhydrous methanol (99.7%) were obtained from Alfa Aesar (Spain). Methylamine, solution 40% in water, synthesis grade was purchased from Scharlab, Spain. Al the materials were used as received.

Methods:

Synthesis of the methylammonium manganese (II) bromide organic – inorganic halides. Gram scale synthesis of the methylammonium manganese (II) bromide halide materials was done in two steps. In the first step, we synthesized the methylammonium bromide (MABr) salt, while in the second one different amounbts of MABr were mixed with $MnBr_2•4H_2O$ to produce the halide $MABr_xMnBr$ with different molar ratios (x).

Synthesis of methylammonium bromide *(CH_3NH_3Br, MABr):* methyl amine (0.5 mL, 0.25 M) was first added to 10 mL of ethyl acetate in a round bottom flask and then 2.5 mL of hydrobromic acid were added dropwise under stirring at 0-2 °C for two hours. The transparent homogeneous CH_3NH_3Br solution was then subjected to evaporation at 60 °C in a rotary evaporator until slightly yellow solid was obtained. The solid was then washed with ethyl acetate and dried again in a rotary evaporator at 40 °C to get a pure, white crystalline solid of CH_3NH_3Br. The washing procedure was repeated thrice to ensure the removal of excess HBr.

Synthesis of $MABr_xMnBr$ halides:

To synthesize the $MABr_xMnBr$ halides, the molar ratio of MABr and MnBr was varied from 0.5 to 2.0 in comparison to the Mn^{2+} concentration. In general, different amounts of MABr (**Table S1**) were dissolved in 5 mL of methanol with a fixed amount of $MnBr_2•4H_2O$ (0.2M, 0.284 g). The solution was then subjected to slow evaporation at 40°C for several days until red or yellow/green emissive solid was obtained. The color of the obtained solids depends on the molar ratio between the two salts (**Table S1**). Consequently, the obtained solids were dried overnight in an oven at 120 °C.

Synthesis of single crystals: The single crystals of **2** were synthesized by a slow evaporation technique at room temperature. As a typical experiment process, 0.300 g of methylammonium bromide and 0.284 g of manganese (II) bromide tetrahydrate were dissolved in 10 ml of distilled water. The resulting solution was then acidified with 4 mL of concentrated HBr under constant magnetic stirring for 3 h until a clear solution was obtained. The solution was kept at a

temperature of 45 °C in an oven and after slow evaporation for 3 days single crystals of **2** were formed.

Preparation of the paper stripes for vapochromism studies:

Scheme S1 shows the detailed procedure for the preparation of the stripes for the vaprochromic studies. In **Step 1** the filter papers were soaked in the parent MeOH solution containing 0.24 g of MABr and 0.284 g of $MnBr_2•4H_2O$. The paper strip is then dried and activated 55°C (328 K) (**Step 2**). Under UV light the activated paper stripe shows green emission at 328 K and following a cooling down to RT (298 K) the emission becomes yellow in dry atmosphere (**Step 3**). In **Step 4** the paper stripe is exposed to a saturated atmosphere of the solvent vapors for 30 to 50 min. If interaction is observed the activated paper stripe loses its yellow color under UV light. The paper stripe that has shown reaction (loss of emission) can be re-activated (**Step 6**) by heating to 328 K (green emission under UV light) and can be re-used following a cool-down to RT (**Step 6**).

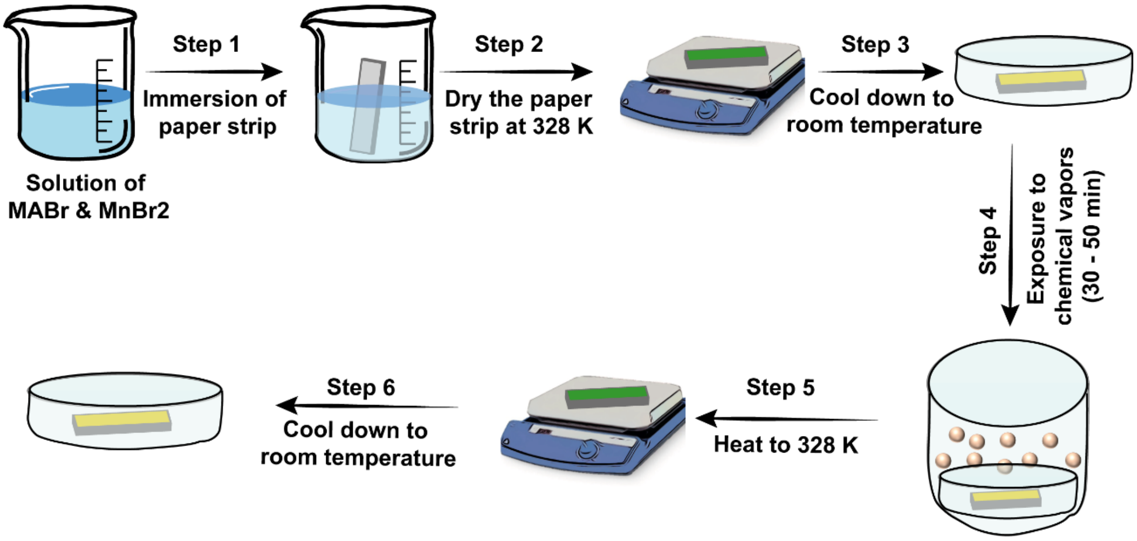

Scheme 1. Illustration of the man steps in the vapochromic studies. The color of the paper stripe indicates the emission color under UV light (365 nm).

Characterizations and measurements

For the single crystal X-ray measurements, a suitable crystal was selected and placed on a MiTeGen micromount on an XtaLAB Synergy R, HyPix-Arc 100 diffractometer. The crystal was kept at a steady T = 250 K during data collection. The structure was solved with the SHELXT 2018/2 [1] structure solution program using the Intrinsic Phasing solution method and by using Olex2 as the graphical interface. [2] The model was refined with version 2018/3 of SHELXT 2018/344 using Least Squares minimization. All non-hydrogen atoms were refined anisotropically. Hydrogen atom positions were calculated geometrically and refined using the riding model. Important structural refinement parameters are summarized in **Table S2-S8** and

the crystallographic data are also supplied. The DIAMOND program was used for the crystal structure plotting. The crystallographic data are deposited in the Cambridge Crystallographic Data Centre (CCDC 2323500 and CCDC 2339285). The data can be downloaded from the site (www.ccdc.cam.ac.uk/data_request/cif). The Hirshfeld surfaces and the 2D finger plots were generated by the program Crystal Explorer 3.1 based on the CIF files. [3]

The powder X-ray diffraction (PXRD) pattern of the synthesized perovskites was carried out using a PANalytical diffractometer (X'Pert Pro model) and an X Bruker D8 Advance. The conditions used were 45 kV, 40 mA, CuKα radiation, and a system of slits (soller-mask-divergence-antiscatter) of 0.04 rad-10 mm-1/8◦ -1/4◦ with an X'celerator detector.
Thermogravimetric analysis (TGA) and differential scanning calorimetry (DSC) analysis of synthesized compound (DSC) were undertaken on a SDT Q600 calorimeter under the dynamic Nitrogen (100 mL/min flow rate) in temperature range of 25–900 °C at the scan rate of 10 °C/min. Platinum crucibles were used as containers. The 100°C PXRD patterns were recorded on a Bruker D8 Advance diffractometer operating at 30 kV/20 mA and equipped with CuKα radiation, Vantec-1 PSD detector, Anton Parr HTK2000 high-temperature furnace, and Pt sample holder.

Electron-spin paramagnetic resonance (EPR): EPR spectra were carried out at room temperature using a Bruker ELEXSYS E500 spectrometer operating at the X-band. The spectrometer was equipped with a super-high-Q resonator ER-4123-SHQ, the magnetic field was calibrated by a NMR probe and the frequency inside the cavity (~9.36 GHz) was determined with an integrated MW-frequency counter.

The UV/Vis diffuse reflectance (DR) spectra were recorded on a JASCO V-670 spectrophotometer equipped with a 60 mm integrating sphere unit (JASCOISN-723). The longitudinal axes of the spectrum were converted using the Kubelka–Munk (K-M) function from reflectance (%R) to K-M units $F(R) = ((1 - R)^2(2R)^{-1})$ where R is the diffuse reflectance intensity from the sample. The emission and excitation spectra were measured by a FluoroMax-4 (Jobin-Yvon) spectrofluorometer. Photoluminescence quantum yield (PLQY) measurements were performed using the quanta-ϕ (HORIBA) integrating sphere accessory, attached to the "NanoLog" Horiba Jobin Yvon spectrofluorometer. The temperature-controlled emission spectra measurements were performed with a home build system.

Time-resolved photoluminescence (TR-PL) measurements were performed by exciting the samples with 40 ps-pulsed (<1 mW, 40 MHz repetition rate) diode lasers (PicoQuant, Germany) centered at 371 and 433 nm. The system was equipped with a laser driver (PDL820B, PicoQuant, Germany) for burst operation that allows the measurement of luminescence decays at time windows up to several seconds. The emission decays were collected and analyzed through a time correlated single photon counting (TCSPC) and multi-channel scaling board (TimeHarp260 (nano), PicoQuant, Berlin, Germany). The fluorescence signal was gated at a magic angle (54.7°) and monitored at 90° with respect to the excitation beam at discrete emission wavelengths. The experimental decays were analyzed using an exponential function without re-convolution (tailfit). The decays at different temperature were collected following heating the sample to 100 °C and recording the decays every 20 sec in a period of 3 min. The temperature of the sample at each point as it cooled down was monitored by a thermocouple.

The low temperature steady-state and time-resolved measurements were performed using a variable temperature liquid nitrogen cooled cryostat (OptistatDN, Oxford Instruments, England) connected to ITC601 temperature controller (Oxford instruments, England).

Table S1. Ratio of the different organic (methylammonium bromide, MABr) and inorganic ($MnBr_2$•$4H_2O$) salts used to prepare the studied organic-inorganic Mn-based hybrid materials, and color of the as-synthesized solids under UV light (365 nm) irradiation.

Sample (MABr: $MnBr_2$)	MABr g	$MnBr_2$•$4H_2O$ g	Color (ambient / 25°C)	Color (365 nm / 25°C)	Color (365 nm / 120°C)
**1 (1:2)**[a]	0.06	0.284	Red	red	red
**2 (2:1)**[a]	0.24	0.284	Yellow	yellow	green
**3 (1:1)**[a]	0.12	0.284	Red	red + yellow	red + green

[a]the values in parenthesis indicate the molar ratio MABr:$MnBr_2$

Table S2. Crystal data and structure refinement for **2** at room temperature (2:1 of MABr:$MnBr_2$)

Identification code	CCDC 2323500
Empirical formula	$C_4H_{28}Br_6MnN_4O_2$
Formula weight	698.70
Temperature/K	250.00(10)
Crystal system	Monoclinic
Space group	C2/m
a/Å	12.0487(4)
b/Å	8.8974(4)
c/Å	10.2363(4)
α/°	90
β/°	107.020(4)
γ/°	90
Volume/Å^3	1049.29(7)
Z	2
ρ_{calc}g/cm^3	2.211
μ/mm^{-1}	18.504
F(000)	662.0
Crystal size/mm^3	0.337 × 0.212 × 0.158
Radiation	Cu Kα (λ = 1.54184)
2Θ range for data collection/°	9.034 to 136.226
Index ranges	$-14 \le h \le 10, -10 \le k \le 10, -12 \le l \le 12$
Reflections collected	4836
Independent reflections	1021 [R_{int} = 0.0451, R_{sigma} = 0.0189]
Data/restraints/parameters	1021/0/49
Goodness-of-fit on F^2	1.113
Final R indexes [I>=2σ (I)]	R_1 = 0.0362, wR_2 = 0.1002
Final R indexes [all data]	R_1 = 0.0362, wR_2 = 0.1002
Largest diff. peak/hole / e Å^{-3}	0.88/-1.00

Table S3. Fractional atomic coordinates (×10^4) and equivalent isotropic displacement parameters (Å^2×10^3) for **2** at room temperature. U_{eq} is defined as 1/3 of the trace of the orthogonalized U_{IJ} tensor.

Atom	*x*	*y*	*z*	U(eq)
Br1	2703.9(4)	5000	4773.7(5)	29.9(2)
Br2	4523.6(4)	5000	2257.9(5)	33.3(2)
Br3	4162.7(5)	0	2358.4(6)	40.2(2)
Mn1	5000	5000	5000	21.7(3)
O1	5000	2512(4)	5000	39.4(9)
N1	1911(4)	2261(5)	2476(4)	48.0(9)
C1	1535(4)	2855(6)	1118(4)	47.6(10)

Table S4. Anisotropic displacement parameters (Å^2×10^3) for **2** at room temperature. The anisotropic displacement factor exponent takes the form: $-2\pi^2[h^2a^{*2}U_{11}+2hka^*b^*U_{12}+\ldots]$.

Atom	U_{11}	U_{22}	U_{33}	U_{23}	U_{13}	U_{12}
Br1	21.9(3)	38.4(4)	27.4(3)	0	4.2(2)	0
Br2	31.4(3)	42.2(4)	22.5(3)	0	1.9(2)	0
Br3	45.6(4)	38.5(4)	37.2(4)	0	13.2(3)	0
Mn1	20.4(5)	18.4(5)	24.3(6)	0	3.4(4)	0
O1	54(2)	18.0(18)	39(2)	0	4(2)	0
N1	59(2)	40(2)	41(2)	-3.6(16)	9.3(17)	-8.1(18)
C1	45(2)	59(2)	39(2)	-2(2)	12.5(17)	-1(2)

Table S5. Bond lengths for **2** at room temperature.

Atom	Atom	Length/Å		Atom	Atom	Length/Å
Br1	Mn1	2.7078(5)		Mn1	O1	2.213(3)
Br2	Mn1	2.6953(5)		N1	C1	1.431(6)
Mn1	O1[1]	2.213(3)				

[1]1-X,1-Y,1-Z

Table S6. Bond angles for **2**.

Atom	Atom	Atom	Angle/°		Atom	Atom	Atom	Angle/°
Br1[1]	Mn1	Br1	180.0		O1[1]	Mn1	Br1	90.0
Br2[1]	Mn1	Br1[1]	90.578(16)		O1[1]	Mn1	Br1[1]	90.0
Br2	Mn1	Br1	90.578(16)		O1	Mn1	Br2[1]	90.0
Br2[1]	Mn1	Br1	89.422(16)		O1[1]	Mn1	Br2	90.0
Br2	Mn1	Br1[1]	89.422(16)		O1[1]	Mn1	Br2[1]	90.0
Br2[1]	Mn1	Br2	180.0		O1	Mn1	Br2	90.0
O1	Mn1	Br1[1]	90.0		O1	Mn1	O1[1]	180.0
O1	Mn1	Br1	90.0					

[1]1-X,1-Y,1-Z

Table S7. Hydrogen bonds for **2** at room temperature.

D	H	A	d(D-H)/Å	d(H-A)/Å	d(D-A)/Å	D-H-A/°
O1	H1A	Br3[1]	0.88	2.55	3.425(2)	173.8
O1	H1B	Br3	0.88	2.55	3.425(2)	173.8
N1	H1C	Br2[2]	0.90	2.59	3.463(4)	162.8
N1	H1D	Br1	0.90	2.43	3.327(4)	174.2
N1	H1E	Br3	0.90	2.52	3.408(4)	168.3
C1	H1G	Br2	0.97	3.10	3.939(5)	145.6
C1	H1H	Br3[3]	0.97	3.08	3.943(5)	148.3

[1]1-X,-Y,1-Z; [2]-1/2+X,-1/2+Y,+Z; [3]-1/2+X,1/2+Y,+Z

Table S8. Hydrogen atom coordinates (Å×10^4) and isotropic displacement parameters (Å^2×10^3) for **2** at room temperature.

Atom	*x*	*y*	*z*	U(eq)
H1A	5180.36	1905.24	5713.4	59
H1B	4819.54	1905.24	4286.7	59
H1C	1345.44	1684.35	2623.23	58
H1D	2069.76	3022.9	3080.54	58
H1E	2553.23	1702.6	2577.53	58
H1F	1343.74	2033.56	466.92	71
H1G	2151.17	3454.94	950.87	71
H1H	853.72	3477.77	1015.45	71

Table S9. Crystal data and structure refinement for **2** (2:1 of MABr:$MnBr_2$) at 80 K.

Identification code	CCDC 2339285
Empirical formula	$C_4H_{28}Br_6MnN_4O_2$
Formula weight	698.70
Temperature/K	81.00 (9)
Crystal system	Monoclinic
Space group	C2/m
a/Å	11.9439(8)
b/Å	8.8470(6)
c/Å	10.1026(7)
α/°	90
β/°	107.126(7)
γ/°	90
Volume/Å^3	1020.18(7)
Z	2
ρ_{calc}g/cm^3	2.275
μ/mm^{-1}	12.395
Crystal size/mm^3	0.36 × 0.24 × 0.10

Radiation	Mo Kα
Θ range for data collection/°	2.913 to 25.450
Reflections collected	9126
Independent reflections	1021 [R_{int} = 0.1179]
Data/restraints/parameters	1021/0/49
Goodness-of-fit on F^2	1.164
Final R indexes [I>=2σ (I)]	R_1 = 0.0533 wR_2 = 0.1305
Final R indexes [all data]	R_1 = 0.0577, wR_2 = 0.1329
Largest diff. peak/hole / e Å^{-3}	1.769/-1.361

Table S10. Fractional atomic coordinates (×10^4) and equivalent isotropic displacement parameters (Å^2×10^3) for **2** at 80 K. U_{eq} is defined as 1/3 of the trace of the orthogonalized U_{ij}.

**Atom**	**x**	**y**	**z**	***Ueq***
C1	1518(6)	2914(10)	1071(8)	19.4(16)
N1	1914(5)	2219(8)	2456(7)	19.3(14)
Br1	2683.6(7)	5000	4757.7(10)	14.0(3)
Br2	4538.7(8)	5000	2228.2(10)	15.0(3)
Mn1	5000	5000	5000	12.4(5)
O1	5000	2515(8)	5000	18.4(16)

Table S11. Anisotropic displacement parameters (×10^4) for **2** at 80 K. The anisotropic displacement factor exponent takes the form: $-2\pi^2[h^2a^{*2} \text{ x } U_{11}+\ldots+2hka^* \text{ x } b^* \text{ x } U_{12}]$.

**Atom**	***U11***	***U22***	***U33***	***U23***	***U13***	***U12***
C1	14(3)	21(4)	24(4)	1(3)	6(3)	3(3)
N1	13(3)	19(3)	25(4)	-2(3)	4(3)	-1(2)
Br1	6.6(5)	19.2(6)	15.5(6)	0	2.2(4)	0
Br2	8.8(5)	21.5(6)	13.8(5)	0	2.0(4)	0
Mn1	4.9(9)	16.4(12)	16.0(11)	0	3.4(8)	0
O1	18(3)	14(4)	23(4)	0	4(3)	0
Br3	12.3(5)	19.8(6)	17.3(6)	0	5.3(4)	0

Table S12. Bond lengths for **2** at 80 K.

**Atom**	**Atom**	**Length/Å**
C1	N1	1.474(10)
Br1	Mn1	2.7048(8)
Br2	Mn1	2.6901(10)
Mn1	O1[1]	2.198(7)
Mn1	O1	2.198(7)

[1]1-X,1-Y,1-Z

Table S13. Bond angles for **2** at 80 K.

**Atom**	**Atom**	**Atom**	**Angle/°**		**Atom**	**Atom**	**Atom**	**Angle/°**
Br1	Mn1	Br1[1]	180.0		O1	Mn1	Br1	90.000(1)

Br2	Mn1	Br1	90.88(3)		O1[1]	Mn1	Br2	90.0
Br2[1]	Mn1	Br1[1]	90.88(3)		O1	Mn1	Br2[1]	90.0
Br2	Mn1	Br1[1]	89.12(3)		O1	Mn1	Br2	90.0
Br2[1]	Mn1	Br1	89.12(3)		O1[1]	Mn1	Br2[1]	90.0
Br2	Mn1	Br2[1]	180.0		O1[1]	Mn1	O1	180.0
O1[1]	Mn1	Br1	90.000(1)					
O1[1]	Mn1	Br1[1]	90.000(1)					
O1	Mn1	Br1[1]	90.000(1)					

[1]1-X,1-Y,1-Z

Table S14. Hydrogen bonds for **2** at 80 K.

D	H	A	d(D-H)/Å	d(H-A)/Å	d(D-A)/Å	D-H-A/°
C1	H1C	Br2[1]	0.98	3.14	4.108(8)	170.8
C1	H1D	Br2	0.98	3.06	3.912(7)	145.8
C1	H1E	Br3[2]	0.98	3.00	3.884(7)	150.1
N1	H1F	Br3	0.91	2.45	3.358(6)	173.1
N1	H1G	Br2[3]	0.91	2.52	3.401(6)	162.0
N1	H1H	Br1	0.91	2.41	3.323(7)	177.2
O1	H1A	Br3[4]	0.87	2.53	3.400(5)	174.5
O1	H1B	Br3	0.87	2.53	3.400(5)	174.5

[1]1-X,-Y,1-Z; [2]-1/2+X,-1/2+Y,+Z; [3]-1/2+X,1/2+Y,+Z

Table S15. Hydrogen fractional atomic coordinates (Å×10^4) and equivalent isotropic displacement parameters (Å^2×10^3) for **2** at 80 K. U_{eq} is defined as 1/3 of the trace of the orthogonalized U_{ij}.

Atom	x	y	z	*Ueq*
H1C	1274.92	2121.08	367.47	29
H1D	2160.69	3498.5	905.59	29
H1E	853.58	3586.41	1018.31	29
H1F	2533.49	1599.52	2506.94	23
H1G	1318.22	1671.7	2607.65	23
H1H	2134.95	2955.87	3109.86	23
H1A	5190.53	1909.81	5715.7	28
H1B	4809.47	1909.81	4284.3	28

Table S16. Atomic Occupancies for all atoms that are not fully occupied in **2** at 80 K.

Atom	Occupancy
H1A	0.5
H1B	0.5

Table S17A. Values of time constants (τ_i) and normalized (to 100) pre-exponential factors (a_i) obtained from the fit of the emission decays recorded at the indicated wavelengths (λ_{obs}) and upon excitation (λ_{EX}) at 371 and 433 nm for **1**.

	λ_{EX} = 371 nm				λ_{EX} = 433 nm			
λ_{obs}	τ_1 / µs	a_1 / %	τ_2 / µs	a_2 / %	τ_1 / µs	a_1 / %	τ_2 / µs	a_2 / %
**500 nm**	**7**	84	**62**	14	**6**	43	**66**	57
**530 nm**	**15**	80	**113**	20	**12**	43	**96**	57
**560 nm**	**15**	66	**115**	34	**43**	43	**151**	57
**580 nm**	**56**	54	**148**	46	**61**	53	**161**	47
**620 nm**	**56**	54	**148**	46	**61**	53	**145**	47
**650 nm**	**56**	54	**148**	46	**61**	57	**145**	43
**675 nm**	**56**	53	**148**	47	**61**	57	**145**	43
**725 nm**	**56**	53	**148**	47	**61**	58	**145**	42

Table S17B. Values of time constants (τ_i) and normalized (to 100) pre-exponential factors (a_i) obtained from the fit of the emission decays recorded at the indicated wavelengths (λ_{obs}) and upon excitation (λ_{EX}) at 371 and 433 nm for **2**.

	λ_{EX} = 371 nm				λ_{EX} = 433 nm			
λ_{obs}	τ_1 / µs	a_1 / %	τ_2 / µs	a_2 / %	τ_1 / µs	a_1 / %	τ_2 / µs	a_2 / %
**475 nm**	**15**	86	**145**	14	**13**	90	**145**	9
**500 nm**	**15**	84	**145**	16	**13**	86	**145**	14
**530 nm**	**15**	84	**145**	16	**13**	86	**145**	14
**560 nm**	**15**	80	**145**	20	**13**	86	**145**	14
**580 nm**	**15**	55	**145**	45	**13**	59	**145**	41
**620 nm**	**15**	-100	**148**	100	**11**	-100	**147**	100
**650 nm**	**15**	-100	**148**	100	**11**	-100	**147**	100
**675 nm**	**15**	-100	**148**	100	**11**	-100	**147**	100
**725 nm**	**15**	-100	**148**	100	**11**	-100	**147**	100

Table S17C. Values of time constants (τ_i) and normalized (to 100) pre-exponential factors (a_i) obtained from the fit of the emission decays recorded at the indicated wavelengths (λ_{obs}) and upon excitation (λ_{EX}) at 371 and 433 nm for **3**.

	λ_{EX} = 371 nm				λ_{EX} = 433 nm			
λ_{obs}	τ_1 / µs	a_1 / %	τ_2 / µs	a_2 / %	τ_1 / µs	a_1 / %	τ_2 / µs	a_2 / %
**475 nm**	**17**	80	**145**	20	**16**	90	**141**	10
**500 nm**	**17**	78	**145**	22	**16**	88	**141**	12
**530 nm**	**17**	78	**145**	22	**16**	88	**141**	12
**560 nm**	**17**	75	**145**	25	**16**	80	**141**	20
**580 nm**	**24**	62	**140**	38	**35**	47	**152**	53
**620 nm**	**34**	56	**135**	44	**55**	51	**141**	49
**650 nm**	**34**	54	**135**	46	**55**	48	**141**	52
**675 nm**	**34**	54	**135**	46	**55**	46	**141**	54
**725 nm**	**34**	54	**135**	46	**55**	38	**141**	62

Table S18. Values of time constants (τ_i) and normalized (to 100) pre-exponential factors (a_i) obtained from the best fit of the emission decays recorded at selected wavelengths and temperatures upon excitation of **1** at 371 nm.

	λ_{Obs} / nm					
	**625**		**675**		**725**	
**T / K**	τ_1 / µs (a_1)	τ_2 / µs (a_2)	τ_1 / µs (a_1)	τ_2 / µs (a_2)	τ_1 / µs (a_1)	τ_2 / µs (a_2)
**77**	-	**423** (100)	-	**428** (100)	-	**440** (100)
**100**	-	**433** (100)	-	**428** (100)	-	**440** (100)
**120**	-	**400** (100)	-	**400** (100)	-	**410** (100)
**140**	-	**363** (100)	-	**367** (100)	-	**370** (100)
**160**	-	**329** (100)	-	**333** (100)	-	**337** (100)
**180**	**126** (19)	**324** (81)	**142** (22)	**326** (78)	**150** (23)	**329** (77)
**200**	**107** (27)	**300** (73)	**106** (26)	**299** (74)	**126** (29)	**307** (71)
**220**	**97** (33)	**269** (67)	**119** (40)	**281** (60)	**114** (38)	**279** (62)
**240**	**79** (37)	**231** (63)	**83** (36)	**232** (64)	**88** (41)	**237** (59)
**260**	**71** (44)	**200** (56)	**66** (41)	**198** (59)	**71** (43)	**201** (57)
**280**	**62** (44)	**171** (56)	**62** (44)	**175** (56)	**61** (44)	**174** (56)
**298**	**50** (47)	**156** (53)	**55** (46)	**158** (54)	**55** (46)	**160** (54)

Table S19. Values of time constants (τ_i) and normalized (to 100) pre-exponential factors (a_i) obtained from the fit of the emission decays of **1** recorded at 650 nm (λ_{obs}) and upon excitation (λ_{EX}) at 371 nm.

T / K	τ_1 / μs	a_1 / %	τ_2 / μs	a_2 / %
**298**	**53**	55	**134**	45
**315**	**54**	45	**137**	55
**320**	**52**	34	**133**	66
**329**	**51**	22	**128**	78
**337**	**50**	12	**126**	88
**350**	**47**	10	**120**	90
**363**	**43**	111	**111**	94

Table S20. Values of time constants (τ_i) and normalized (to 100) pre-exponential factors (a_i) obtained from the best fit of the emission decays recorded at selected wavelengths and temperatures upon excitation of **2** at 371 nm.

Table 1B	λ_{Obs} / nm													
	500		520		550		570		600		625		670	
T / K	τ_1 / μs (a_1)	τ_2 / μs (a_2)	τ_1 / μs (a_1)	τ_2 / μs (a_2)	τ_1 / μs (a_1)	τ_2 / μs (a_2)	τ_1 / μs (a_1)	τ_2 / μs (a_2)	τ_1 / μs (a_1)	τ_2 / μs (a_2)	τ_1 / μs (a_1)	τ_2 / μs (a_2)	τ_1 / μs (a_1)	τ_2 / μs (a_2)
**77**	-	**307** (100)	**130** (37)	**360** (63)	-	**370** (100)	**190** (-100)	**472** (100)	**220** (-100)	**470** (100)	**250** (-100)	**472** (100)	-	**440** (100)
**100**	-	**307** (100)	**136** (41)	**365** (59)	-	**377** (100)	**181** (-100)	**495** (100)	**221** (-100)	**468** (100)	**169** (-100)	**480** (100)	-	**410** (100)
**120**	-	**312** (100)	**137** (41)	**365** (59)	-	**377** (100)	**181** (-100)	**496** (100)	**189** (-100)	**517** (100)	**214** (-100)	**436** (100)	-	**410** (100)
**140**	**77** (40)	**288** (60)	**81** (40)	**293** (60)	**91** (35)	**317** (65)	**157** (20)	**366** (80)	**85** (-100)	**420** (100)	**88** (-100)	**423** (100)	**105** (-100)	**431** (100)
**160**	**68** (39)	**277** (61)	**70** (54)	**280** (46)	**72** (48)	**287** (52)	**97** (32)	**296** (68)	**82** (-100)	**319** (100)	**79** (-100)	**394** (100)	**91** (-100)	**410** (100)
**180**	**64** (42)	**264** (58)	**59** (42)	**257** (58)	**74** (37)	**261** (63)	**108** (47)	**281** (53)	-	**410** (100)	**83** (-100)	**393** (100)	**110** (-100)	**366** (100)
**200**	**63** (51)	**251** (49)	**61** (46)	**247** (54)	**64** (49)	**251** (51)	**70** (51)	**258** (49)	-	**400** (100)	**73** (-100)	**393** (100)	**103** (-100)	**407** (100)
**220**	**58** (34)	**242** (66)	**54** (33)	**237** (67)	**61** (34)	**241** (66)	**61** (35)	**246** (65)	**50** (36)	**306** (64)	**78** (-100)	**371** (100)	**93** (-100)	**395** (100)

**240**	**55** (37)	**228** (63)	**49** (37)	**225** (63)	**51** (37)	**227** (63)	**53** (36)	**230** (64)	**49** (34)	**275** (66)	-	-	**75** (-100)	**330** (100)
**260**	**40** (31)	**164** (69)	**43** (34)	**165** (66)	**46** (34)	**167** (66)	**48** (34)	**169** (66)	**33** (24)	**193** (76)	-	-	**62** (-100)	**217** (100)
**280**	**34** (68)	**91** (32)	**25** (51)	**75** (49)	**28** (72)	**80** (28)	**35** (74)	**102** (26)	**33** (44)	**141** (56)	**44** (-100)	**149** (100)	**43** (-100)	**154** (100)
**298**	**16** (81)	**139** (19)	**15** (80)	**134** (20)	**16** (80)	**142** (20)	**15** (85)	**140** (15)	**17** (37)	**143** (63)	**15** (-100)	**141** (100)	**14** (-100)	**142** (100)

Table S21. Values of time constants (τ_i) and normalized (to 100) pre-exponential factors (a_i) obtained from the fit of the emission decays of **2** recorded at 530 nm (λ_{obs}) and upon excitation (λ_{EX}) at 371 nm.

T / K	τ_1 / µs	a_1 / %	τ_2 / µs	a_2 / %
**298**	**15**	55	**125**	45
**313**	**12**	45	**123**	55
**318**	**10**	34	**125**	66
**323**	**10**	22	**130**	78
**328**	**9**	12	**126**	88
**333**	**8**	10	**120**	90
**338**	**-**	-	**125**	94
**350**	**-**	-	**127**	100
**363**	**-**	-	**130**	100

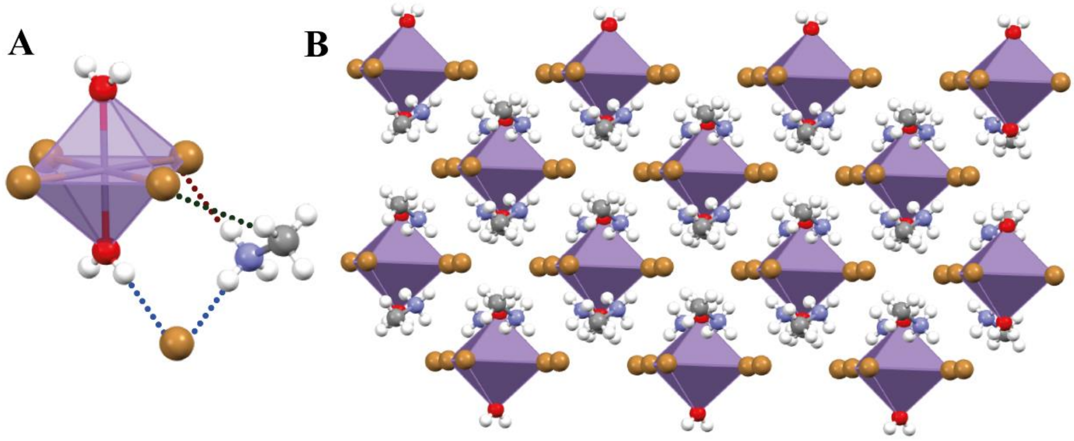

Figure S1. Illustration of the crystalline structure of asymmetric part of the unit cell (**A**) and the packed structure (**B**) for $(MA)_4Br_6Mn(H_2O)_2$ (**2**) at 80K. The dotted lines in **A** indicate the H-bonding interactions between the methylammonium (MA) and Br (free and coordinated with Mn) and between the H atom of water and the uncoordinated Br. The crystallographic data for $MA)_4Br_6Mn(H_2O)_2$ are deposited in the Cambridge Crystallographic Data Centre (CCDC 2339285).

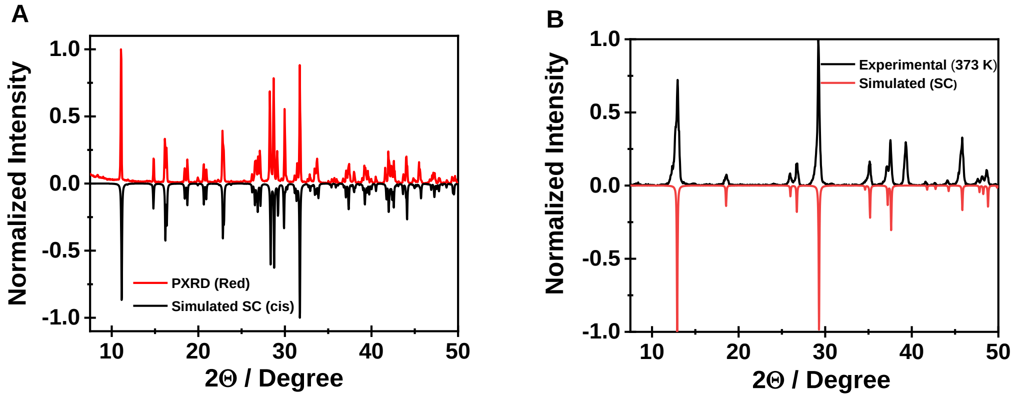

Figure S2. Powder X-ray diffractograms for $MAMnBr_3$ (**1**, ratio 1:2 ($MABr:MnBr_2$)) at **A)** 293 K and **B)** 373 K. The simulated single crystal (SC) diffractograms are shown with negative sign and are recovered from data published in ref. [4]

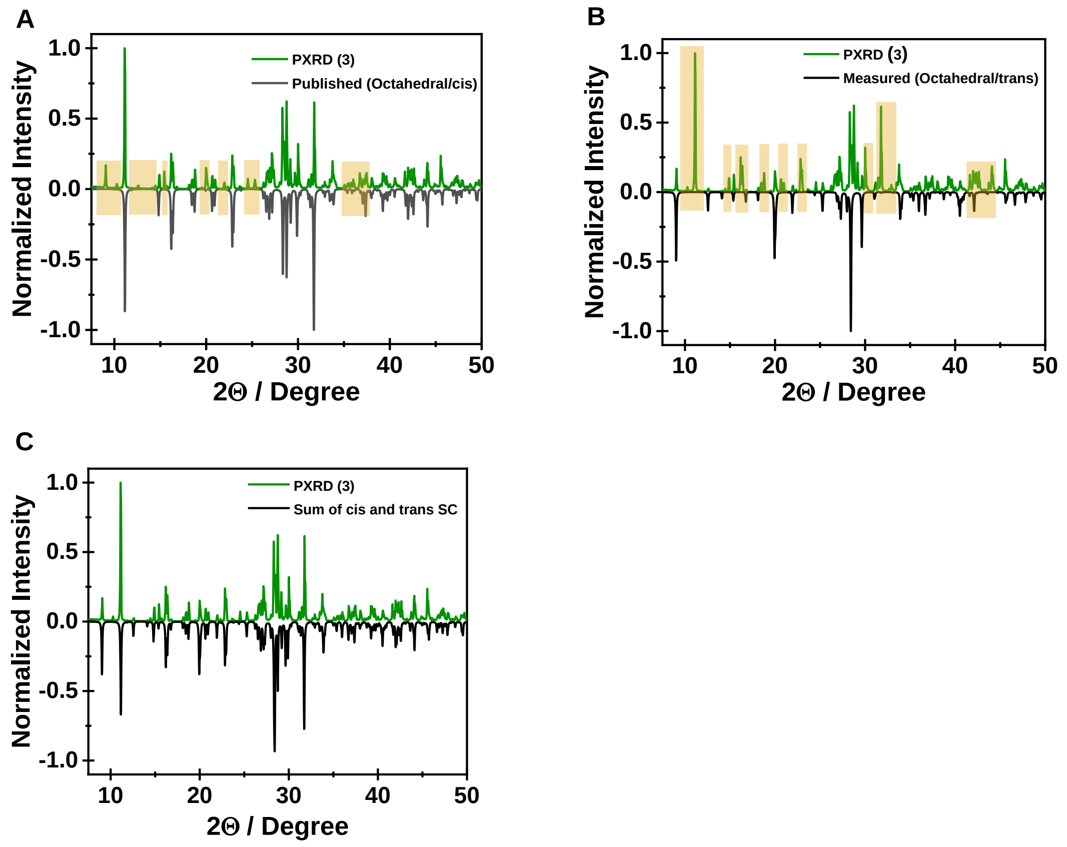

Figure S3. Powder X-ray diffractograms for **3** at room temperature. The shaded areas highlight the differences with the simulated single crystal (SC) diffractograms. The simulated diffractograms are shown with negative sign are recovered from data published in ref. [4]

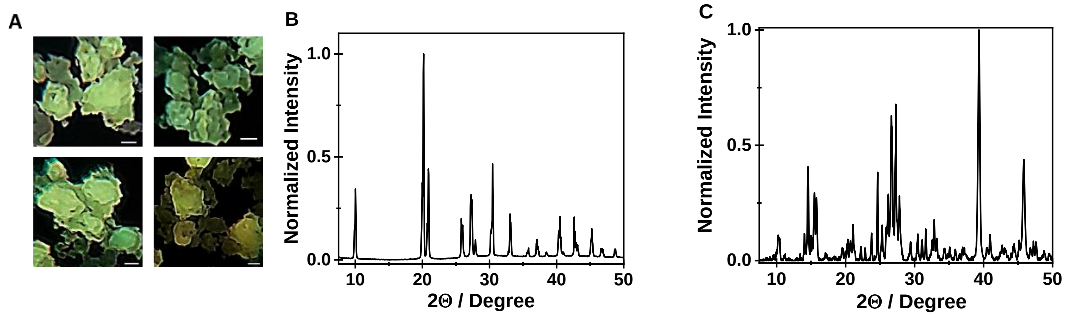

Figure S4. (A) Images of **2** taken with a low-resolution home-built total internal reflection fluorescence (TIRF) microscope (the size of the scale bar is 100 μm). **(B)** PXRD diffractogram of pure methylammonium bromide (MABr) and **(C)** PXRD for **2** at 373 K.

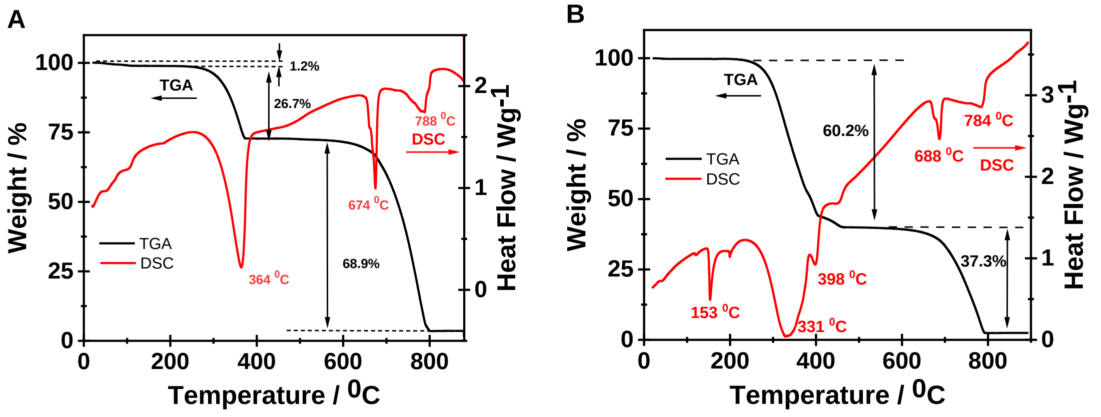

Figure S5. Thermogravimetric (TGA, black) and differential scanning calorimetry (DSC, red) results for **1 (A)** and **2 (B)**.

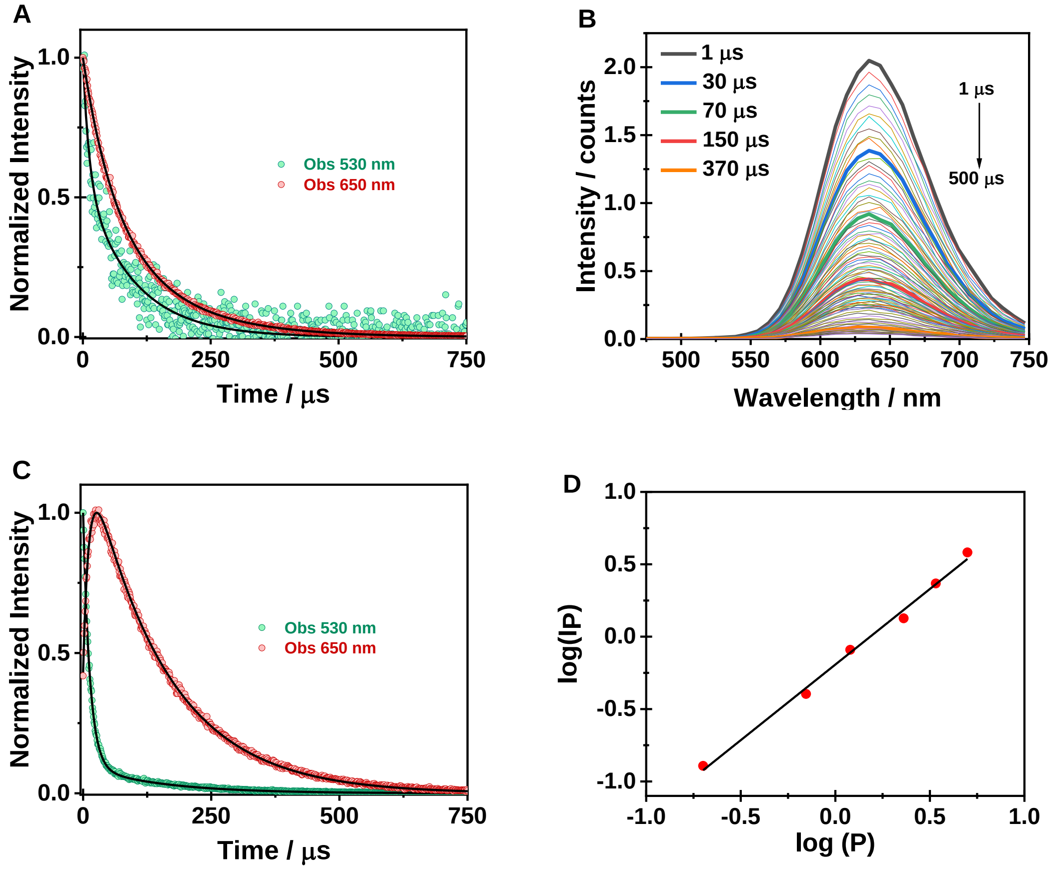

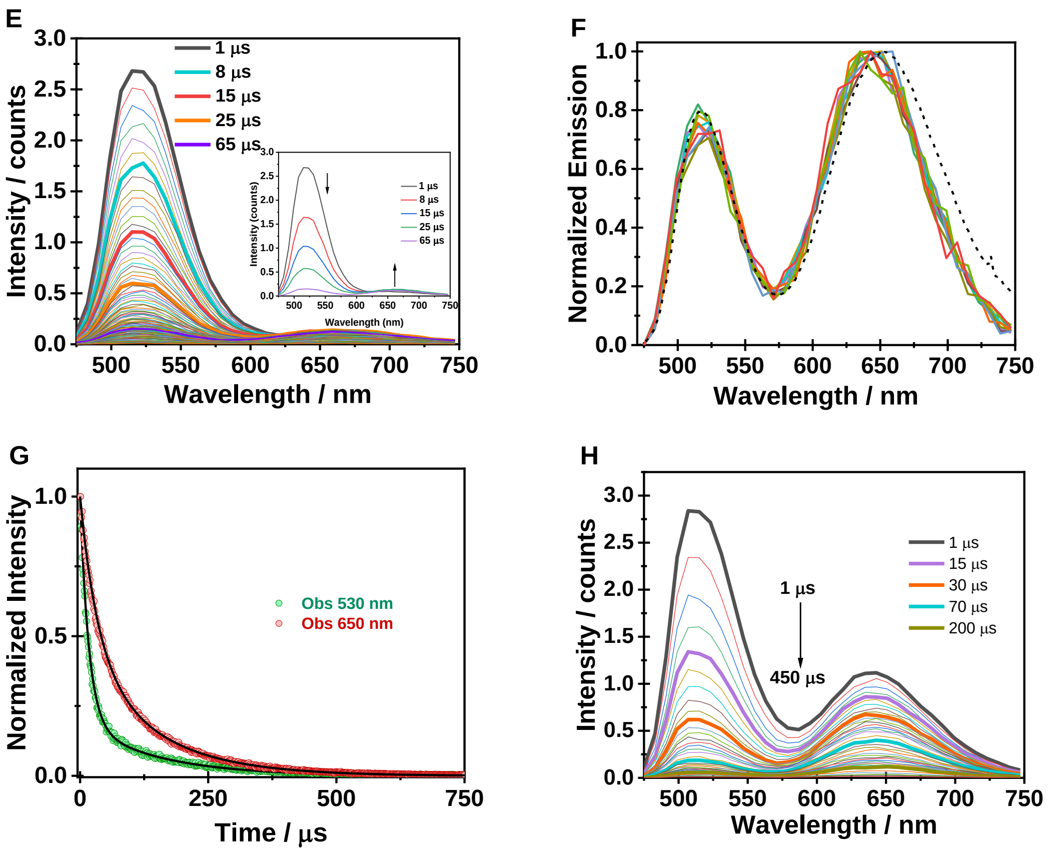

Figure S6. Emission decays collected at 530 and 650 nm (**A, C** and **G**) and time-resolved emission spectra (**B, E** and **H**) following excitation of **1**, **2**, and **3** at 433 nm , respectively. The inset in panel **E** shows selected TRES to highlight the iso-emissive point at 620 nm. **D** shows the dependence of the maximum emission intensity of **2** at 650 nm with the excitation power. **F** shows the normalized TRES of **3** collected at delay times between 100 and 500 μs. The dashed spectrum in **E** corresponds to the steady-state emission one.

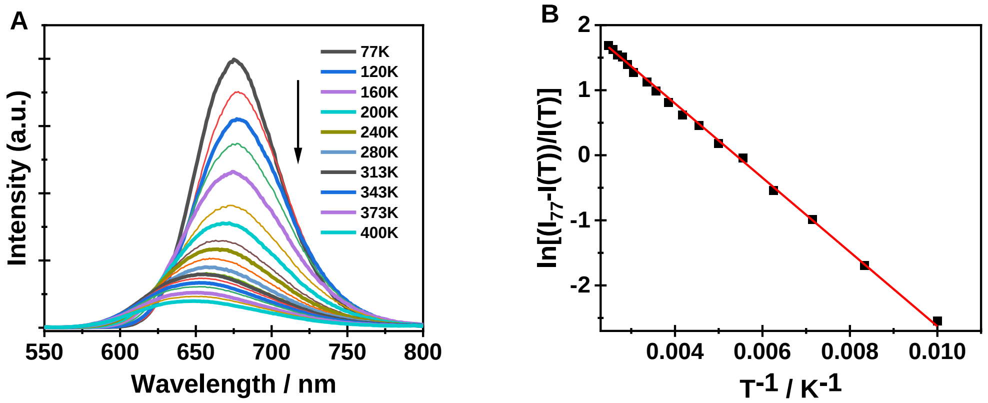

Figure S7. Temperature dependence of the (**A**) emission spectrum of **1** and **(B)** its maximum of emission intensity at 640 nm (λ_{ex} = 360 nm), collected at the indicated temperatures. The solid line in **(B)** is the best fit using Equation 2 in the main text (R^2 = 1.0).

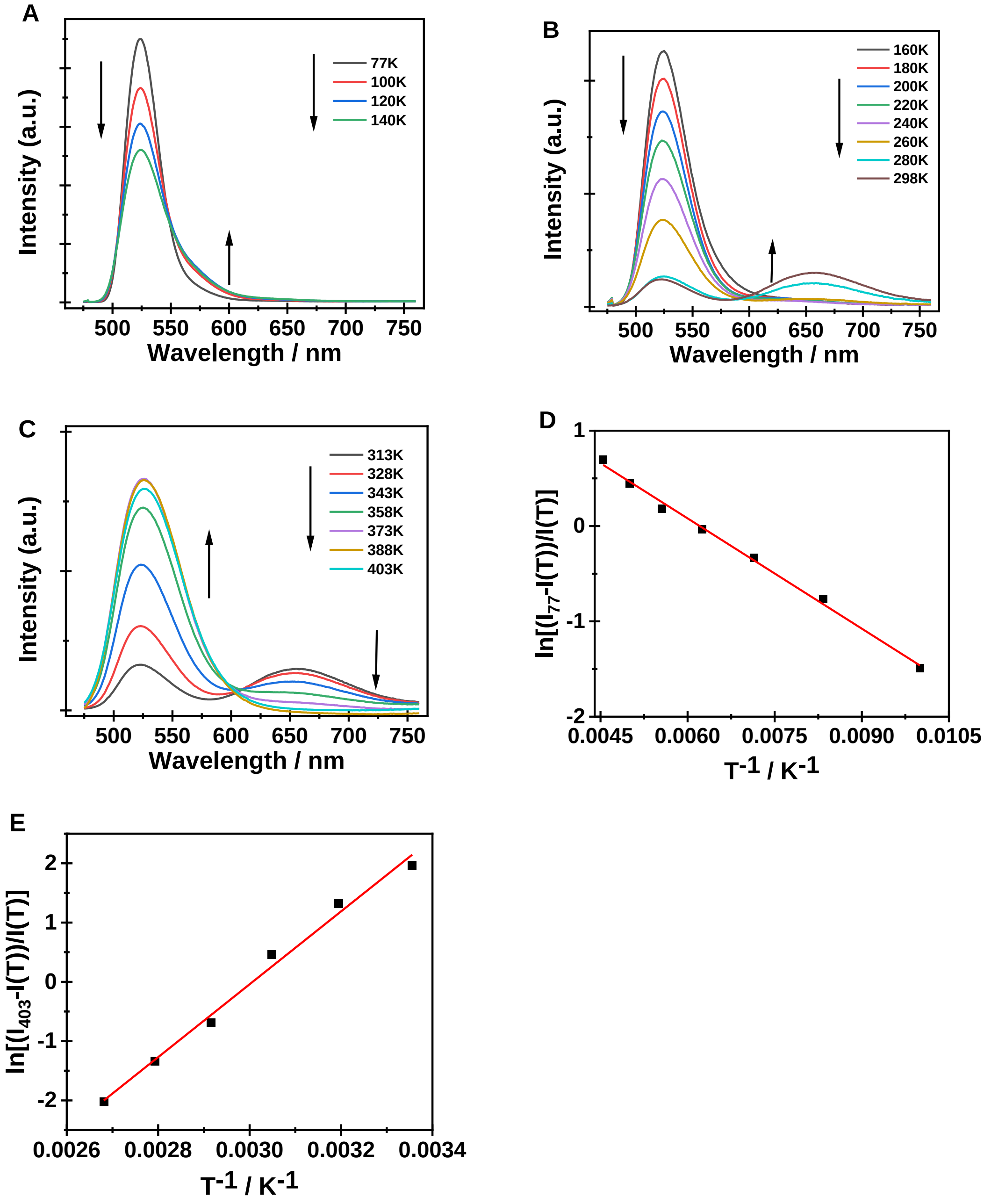

Figure S8. Temperature dependence of the emission spectrum of **2** in the temperature interval of **(A)** 77K – 140K**, (B)** 160K – 298K and **(C)** 313K – 403K. **(D)** and **(E)** represent the maximum of emission intensity at 530 nm (λ_{ex} = 360 nm), collected at 100K-220K and 298K-403K respectively. The solid line in **(D)** and **(E)** is the best fit using Equation 2 in the main text.

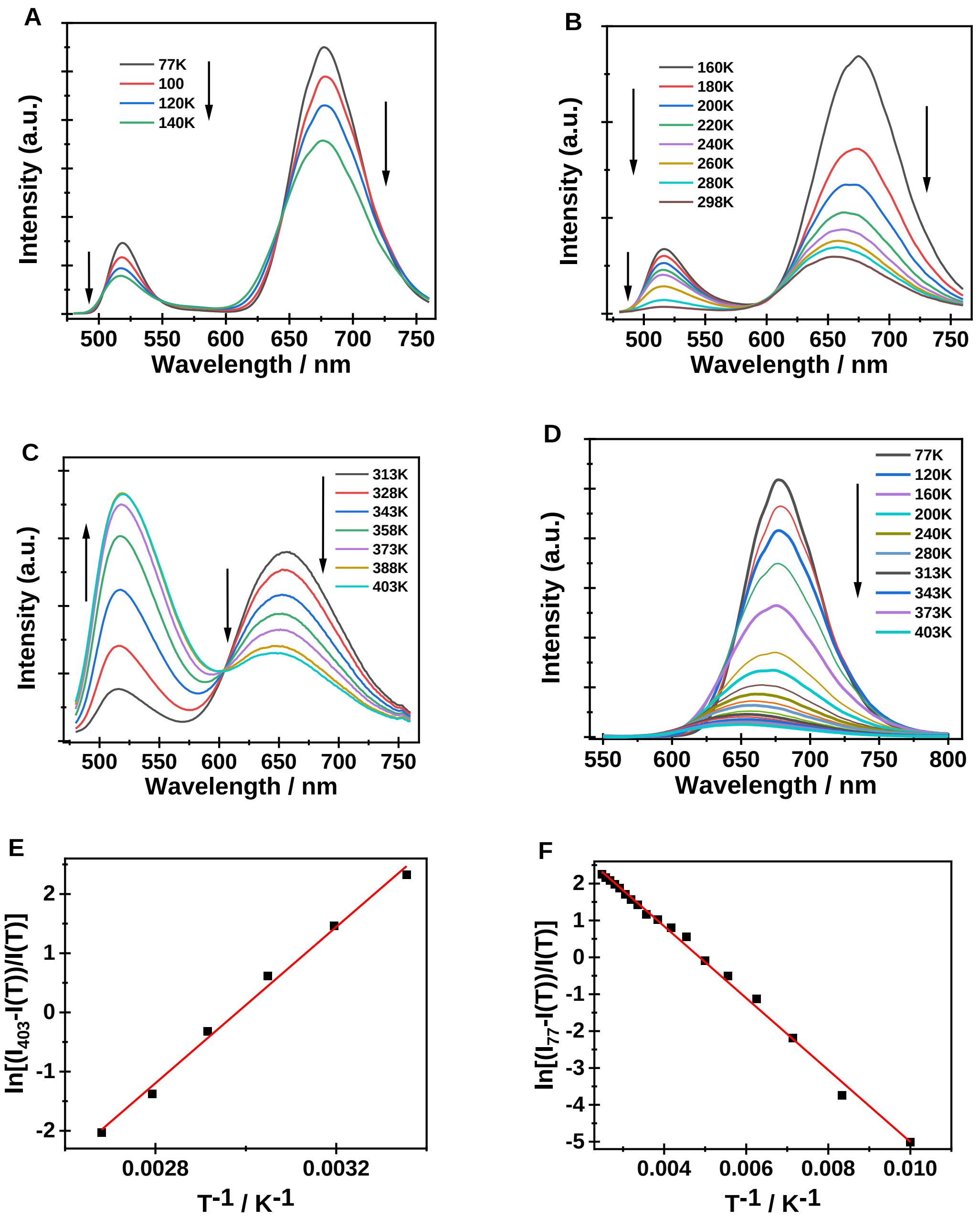

Figure S9. Temperature dependence of the emission spectrum (λ_{ex} = 360 nm) of **3** in the temperature interval of **(A)** 77K – 140K**, (B)** 160K – 298K and **(C)** 313K – 403K. **(D)** represents the maximum of emission intensity at 530 nm, collected at 298K-403K. **(E)** Temperature dependence of emission spectrum of **3** following excitation at 530 nm. **(F)** Temperature dependence of the maximum of **3** emission intensity at 650 nm and λ_{EX} = 530 nm. The solid line in **(D)** and **(F)** is the best fit using Equation 2 in the main text.

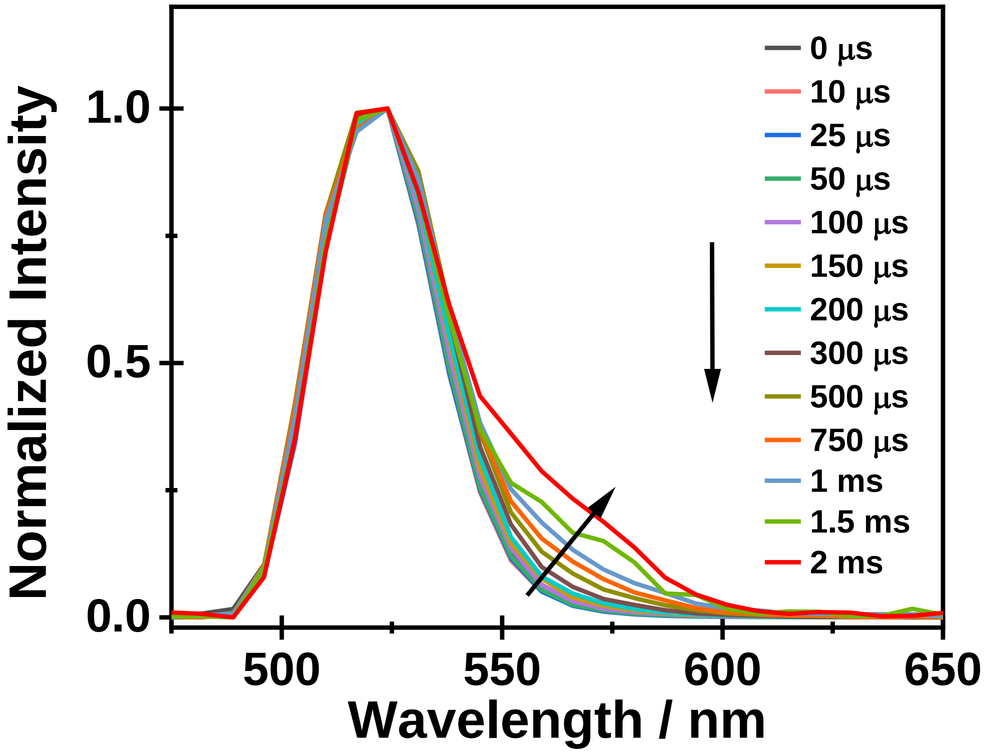

Figure S10. Normalized to the maximum of emission intensity time-resolved emission spectra of 2 collected at 77K following excitation at 371 nm.

Acknowledgments

This work was supported by PID2020-116519RB-I00 and TED2021–131650B-I00 funded by MCIN/AEI/10.13039/501100011033 (Spain) and the European Union (EU); SBPLY/19/180501/000212 and SBPLY/21/180501/000108 funded by JCCM and the EU through "Fondo Europeo de Desarrollo Regional" (FEDER); and 2022-GRIN-34325 funded by UCLM (FEDER). S.R. acknowledges Marie Skłodowska-Curie Actions (project H2020-MSCA-IF-2019-897030, European Union (EU)). MH thanks MCIN for the FPI fellowship PRE2021-099064 financed by MCIN/AEI/10.13059/501100011033 and by FSE+. Technical and human support provided by SGIker (UPV/EHU) is gratefully acknowledged.

References

[1] G. Sheldrick, *Acta Crystallographica Section C* **2015**, *71* (1), 3, https://doi.org/doi:10.1107/S2053229614024218.
[2] O. V. Dolomanov, L. J. Bourhis, R. J. Gildea, J. A. K. Howard, H. Puschmann, *J. Appl. Crystallogr.* **2009**, *42* (2), 339, https://doi.org/doi:10.1107/S0021889808042726.
[3] P. R. Spackman, M. J. Turner, J. J. McKinnon, S. K. Wolff, D. J. Grimwood, D. Jayatilaka, M. A. Spackman, *J. Appl. Crystallogr.* **2021**, *54* (3), 1006, https://doi.org/doi:10.1107/S1600576721002910.

[4] M. Daub, I. Ketterer, H. Hillebrecht, *Z. Anorg. Allg. Chem.* **2018**, *644* (5), 280, https://doi.org/https://doi.org/10.1002/zaac.201700357.
